# Glucocorticoid use in acute respiratory failure from pulmonary causes and association with early changes in the systemic host immune response

**DOI:** 10.1186/s40635-024-00605-y

**Published:** 2024-03-05

**Authors:** Nameer Al-Yousif, Seyed M. Nouraie, Matthew J. Broerman, Yingze Zhang, Tomeka L. Suber, John Evankovich, William G. Bain, Georgios D. Kitsios, Bryan J. McVerry, Faraaz A. Shah

**Affiliations:** 1 Division of Pulmonary, Critical Care, and Sleep Medicine, MetroHealth Medical Center, Cleveland, OH USA; 2 Division of Pulmonary, Allergy, Critical Care, and Sleep Medicine, University of Pittsburgh School of Medicine, 3459 Fifth Avenue, UPMC Montefiore NW 628, Pittsburgh, PA 15213 USA; 3 Acute Lung Injury and Infection Center, University of Pittsburgh School of Medicine, 3459 Fifth Avenue, UPMC Montefiore NW 628, Pittsburgh, PA 15213 USA; 4 Veteran’s Affairs Pittsburgh Healthcare System, Pittsburgh, PA USA; 5 Center for Medicine and the Microbiome, University of Pittsburgh School of Medicine, 3459 Fifth Avenue, UPMC Montefiore NW 628, Pittsburgh, PA 15213 USA; 6 Aging Institute, University of Pittsburgh School of Medicine, 3459 Fifth Avenue, UPMC Montefiore NW 628, Pittsburgh, PA 15213 USA

**Keywords:** Glucocorticoids, ARDS, Pneumonia, Cytokines, Inflammation, Phenotypes, Immune responses

## Abstract

**Background:**

Glucocorticoids are commonly used in patients with or at-risk for acute respiratory distress syndrome (ARDS), but optimal use remains unclear despite well-conducted clinical trials. We performed a secondary analysis in patients previously enrolled in the Acute Lung Injury and Biospecimen Repository at the University of Pittsburgh. The primary aim of our study was to investigate early changes in host response biomarkers in response to real-world use of glucocorticoids in patients with acute respiratory failure due to ARDS or at-risk due to a pulmonary insult. Participants had baseline plasma samples obtained on study enrollment and on follow-up 3 to 5 days later to measure markers of innate immunity (IL-6, IL-8, IL-10, TNFr1, ST2, fractalkine), epithelial injury (sRAGE), endothelial injury (angiopoietin-2), and host response to bacterial infections (procalcitonin, pentraxin-3). In our primary analyses, we investigated the effect of receiving glucocorticoids between baseline and follow-up samples on host response biomarkers measured at follow-up by doubly robust inverse probability weighting analysis. In exploratory analyses, we examined associations between glucocorticoid use and previously characterized host response subphenotypes (hyperinflammatory and hypoinflammatory).

**Results:**

67 of 148 participants (45%) received glucocorticoids between baseline and follow-up samples. Dose and type of glucocorticoids varied. Regimens that used hydrocortisone alone were most common (37%), and median daily dose was equivalent to 40 mg methylprednisolone (interquartile range: 21, 67). Participants who received glucocorticoids were more likely to be female, to be on immunosuppressive therapy at baseline, and to have higher baseline levels of ST-2, fractalkine, IL-10, pentraxin-3, sRAGE, and TNFr1. Glucocorticoid use was associated with decreases in IL-6 and increases in fractalkine. In exploratory analyses, glucocorticoid use was more frequent in participants in the hyperinflammatory subphenotype (58% vs 40%, *p =* 0.05), and was not associated with subphenotype classification at the follow-up time point (*p =* 0.16).

**Conclusions:**

Glucocorticoid use varied in a cohort of patients with or at-risk for ARDS and was associated with early changes in the systemic host immune response.

**Supplementary Information:**

The online version contains supplementary material available at 10.1186/s40635-024-00605-y.

## Background

Glucocorticoids are commonly used in patients with acute respiratory distress syndrome (ARDS) or at-risk for ARDS with proposed mechanisms including reduction of local lung inflammation and dampening of systemic immune responses [1–5]. Clinical trials of glucocorticoids in patients with ARDS or at-risk from a pulmonary infection have had mixed results with some studies suggesting benefit and others showing no beneficial effects (Additional file 1: Table S1) [6–9]. Notably, the glucocorticoid agent, dose, and duration has varied widely between studies increasing the challenge of interpreting discordant findings [10]. Renewed attention to glucocorticoids has resulted from the Coronavirus disease 2019 (COVID-19) pandemic where consistent benefits were observed in patients presenting with moderate to severe disease in randomized clinical trials [11–16] and from differing results from the ESCAPe [6] and CAPE-COD [7] trials testing glucocorticoids in patients with severe pneumonia.

Recent studies have highlighted heterogeneity in patients presenting with ARDS [17]. Two ARDS subphenotypes (so-called hypoinflammatory and hyperinflammatory subphenotypes) have been discovered that differ in the systemic host immune response, prognosis, and potentially response to treatment [18]. Studies from our group and others have demonstrated that hyper- and hypoinflammatory subphenotypes that associate with clinical outcomes exist not only in ARDS cohorts but also in patients at-risk for ARDS [19, 20]. Greater understanding of the effects of glucocorticoids on the systemic immune response in acute respiratory failure may thereby help to elucidate pathways relevant to treatment responsiveness.

The primary aim of our study was to investigate early changes in host response biomarkers in response to real-world use of glucocorticoids in patients with acute respiratory failure who may receive glucocorticoids due to ARDS or who are at-risk due to a pulmonary insult. The secondary aims of our study were to explore associations between glucocorticoid use and previously characterized host response subphenotypes.

## Methods

### Overview

The primary aim of this study was to investigate early longitudinal changes in the systemic host response following clinician-guided receipt of glucocorticoids in ARDS and in patients at-risk for ARDS from a pulmonary insult. In our primary analysis, we used doubly robust inverse probability of treatment weighting (IPTW) [21] to investigate the association of receipt of at least one dose of glucocorticoids with changes in ten host response biomarkers, and we performed sensitivity analyses to ensure consistent results using alternate approaches. In exploratory analyses, we investigated associations of receipt of glucocorticoids with previously described host response subphenotypes [22]. In addition, we performed analyses comparing patients with ARDS and patients at-risk from a pulmonary insult, immunosuppressed and non-immunosuppressed patients, and we performed descriptive analyses of patterns of glucocorticoid use to provide context for our primary results.

### Study population

Participants enrolled prospectively from 2014 to 2020 in the Acute Lung Injury Registry and Biospecimen Repository (ALIR) at the University of Pittsburgh [20]. Briefly, the ALIR is a cohort of mechanically ventilated patients (aged 18–90) with acute respiratory failure within the UPMC Health System in Pittsburgh, Pennsylvania. Informed consent is obtained from patients or their legally authorized representatives under study protocol STUDY19050099 approved by the University of Pittsburgh Institutional Review Board. Exclusion criteria include inability to obtain informed consent, presence of a tracheostomy, pre-existing chronic respiratory failure due to neuromuscular disease, or mechanical ventilation for greater than 72 h.

Participants are classified by consensus of at least three board-certified intensivists into subgroups of (i) ARDS according to the Berlin definition [23]; (ii) at-risk for ARDS by presence of ARDS risk factors (including pulmonary or non-pulmonary sepsis) but not meeting Berlin definition criteria, and (iii) not at-risk when no risk factors are present and chest radiographs appear normal (see Additional file 1 for additional details). Plasma samples are collected upon enrollment within 48 h of intubation (study day 1), at early- (study day 3–5) and late- (study day 7–10) follow-up time points, and are stored at – 80 °C for later analysis [22].

In this study, we included patients with ARDS and patients at-risk for ARDS from a direct pulmonary insult including pneumonia or aspiration. We selected this population for our study because (1) all included patients would have some component of lung injury; and (2) we hypothesized the reasons for administering glucocorticoids to patients with ARDS and to patients at-risk from a pulmonary insult would be similar (to prevent progression of lung injury and improve survival), but patients at-risk for ARDS from non-pulmonary insults or those intubated for airway protection would receive glucocorticoids for different reasons. We included patients that had plasma samples collected at both baseline and early follow-up time points to investigate longitudinal changes in host response in the early phase of acute respiratory failure. We excluded patients that did not have both samples available and performed a complete case analysis without imputation for missing data. Additionally, we did not include patients with COVID-19 in this study as prior studies have demonstrated inflammatory profiles differ compared to non-COVID acute respiratory failure [24, 25].

### Clinical data collection

Patients’ demographics (age, sex, race, body mass index [BMI]), comorbidities (including history of immunosuppression, see Online Supplement for additional details), laboratory data, and measures of oxygenation and ventilation were abstracted from the electronic medical record. Modified Sequential Organ Failure Assessment (mSOFA) scores (not including the neurologic component) were calculated at study enrollment as a measure of severity of illness. Time to liberation from mechanical ventilation and survival at 90 days were determined from review of the medical record.

### Glucocorticoids

We recorded all glucocorticoids (dexamethasone, hydrocortisone, methylprednisolone, prednisolone, and prednisone) administered to participants during the hospitalization by oral or intravenous routes including amount and timing of each dose. Glucocorticoid administration in our cohort was at the discretion of treating clinicians and not influenced in any manner by study personnel or by enrollment of patients into the registry. We converted glucocorticoid doses to methylprednisolone equivalent doses prior to incorporation in analyses (Additional file 1: Table S2). We calculated the total amount of glucocorticoids administered to each patient between baseline and follow-up samples and assessed whether any glucocorticoids were administered prior to baseline sample collection.

### Host response biomarkers

We determined plasma levels of markers of innate immune responses (Interleukin [IL]-6, IL-8, IL-10, tumor necrosis factor receptor 1 [TNFR1], suppression of tumorigenicity-2 [ST-2], fractalkine), epithelial injury (soluble receptor for advanced glycation end-products [sRAGE]), endothelial injury (angiopoietin-2 [ang-2]), and host-response to bacterial infections (procalcitonin and pentraxin-3) with a custom Luminex panel (R&D) as previously described [22].

### Classification into host response subphenotypes

Patients were classified at both baseline and follow-up time points into hypoinflammatory and hyperinflammatory host response subphenotypes by using predicted probabilities for subphenotype classifications from a published parsimonious logistic regression model in this cohort utilizing bicarbonate, tumor necrosis factor receptor (TNFR)-1, angiopoietin-2, and procalcitonin [22].

### Statistical analysis

We grouped patients into those that received at least one dose of oral or IV glucocorticoids between baseline and follow-up samples (Glucocorticoids) and those that did not (No Glucocorticoids). We described daily dose and type of glucocorticoids administered to patients in the Glucocorticoids group, and we compared baseline characteristics between Glucocorticoids and No Glucocorticoids groups with Mann–Whitney U or Fisher’s exact test as appropriate. Data are presented as number (percentage) or median (interquartile range [IQR]). Host response biomarkers were log-transformed prior to analysis and were compared at baseline between Glucocorticoid and No Glucocorticoid groups by Mann–Whitney U tests.

In our primary analysis (Model 1), we investigated associations between receipt of at least one dose of glucocorticoids and host response biomarker levels at follow-up by doubly robust inverse probability of IPTW [21]. Propensity scores for the receipt of clinician-guided glucocorticoids were determined using age, history of immunosuppression, history of chronic obstructive lung disease, severity of illness scores (mSOFA), and vasopressor use at baseline as predictors. Variables were selected for inclusion in the development of the propensity score based on clinical reasoning and literature review. Weights were computed for each participant and incorporated in analyses with log-transformed host response biomarker levels at follow-up as the outcome and receipt of any glucocorticoid as the intervention. Percent change associated with the use of glucocorticoids with 95% confidence intervals were calculated for each host response biomarker. P-values reported in our primary analysis and in other analyses of host response biomarkers are unadjusted but were tested for significance after adjusting for multiple comparisons by the method of Simes [26] and considered significant at a false discovery threshold of 0.1. P-values outside of primary and sensitivity analyses are not adjusted for multiple comparisons and findings should be interpreted as exploratory. We performed three sensitivity analyses to ensure robustness of results. First, we tested the association of receipt of at least one dose of glucocorticoids with each host response biomarker in regression analyses with stratification of participants by decile of propensity score (Model 2). Second, recognizing that receipt of glucocorticoids prior to collection of baseline samples may influence trajectories of host response biomarkers, we adjusted for receipt of any glucocorticoids in the day prior to baseline sample collection in doubly robust IPTW (Model 3) and propensity score stratification analyses (Model 4). In exploratory analyses, we determined host response subphenotype classification (hypoinflammatory or hyperinflammatory) at baseline and follow-up time points and explored associations between glucocorticoid use and subphenotype classification at follow-up with Fisher’s exact test. We repeated IPTW and propensity score regression analyses within hypoinflammatory and hyperinflammatory subphenotypes.

Statistical analyses were performed in Stata version 17 (StataCorp LLC, College Station, TX) and GraphPad PRISM version 9 (GraphPad Software LLC, Boston, MA).

## Results

### Characteristics of enrolled patients

**Fig. 1:**
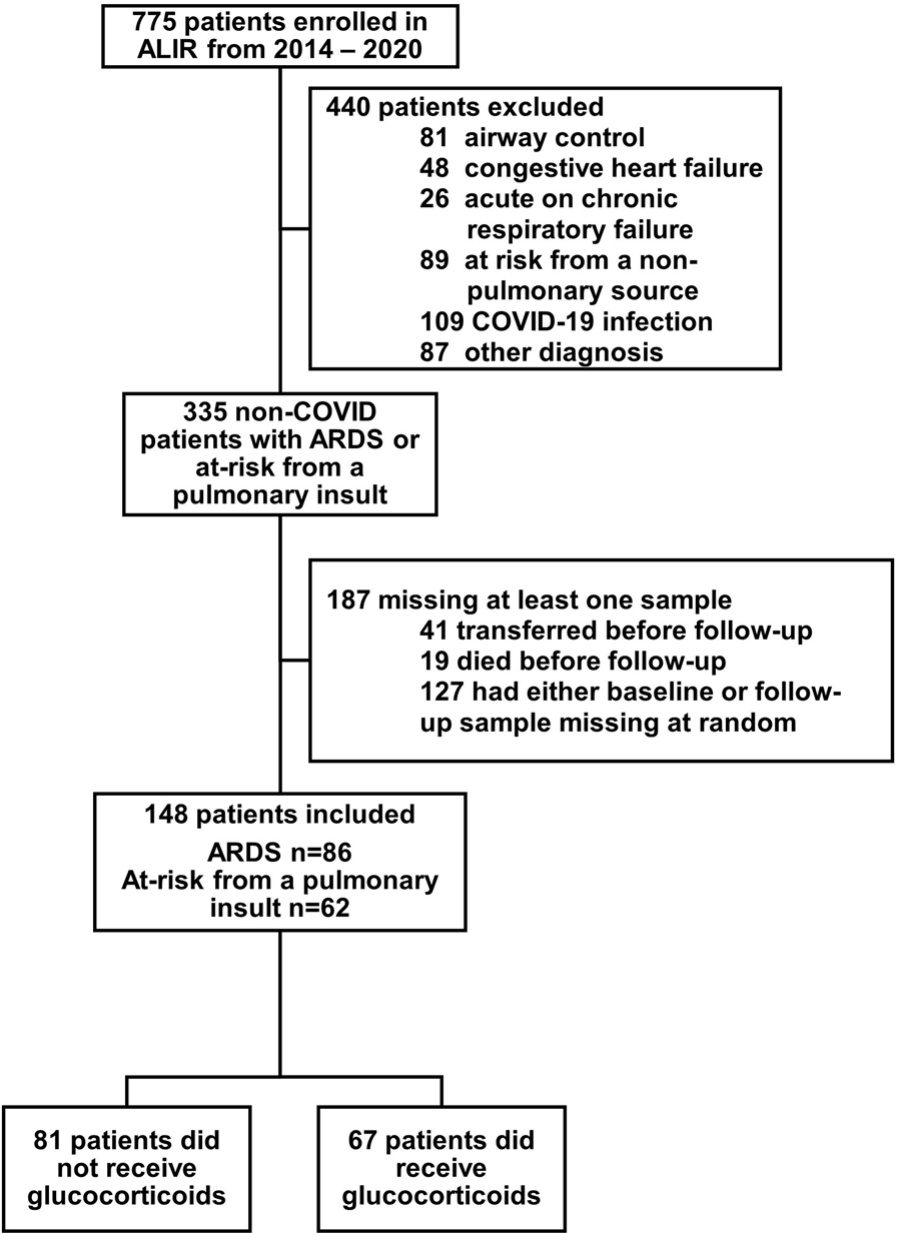
Patient flow diagram. ALIR—Acute Lung Injury Registry and Biospecimen Repository; ARDS—acute respiratory distress syndrome

775 patients were enrolled in ALIR during the study period of whom 86 ARDS and 62 patients at-risk from a pulmonary insult were included in our current study cohort (Fig. 1). Characteristics of patients excluded from the study due to lack of biospecimen availability (transfer before follow-up sample, death before follow-up sample, or missing at random) are included in the Online Supplement (Additional file 1: Table S3). Among patients included in the study, ARDS patients were more likely to be female, have a higher prevalence of immunosuppression, higher PEEP settings on study enrollment, a higher prevalence of sepsis, higher baseline fractalkine and sRAGE, and lower ST-2 levels compared to at-risk patients. (Additional file 1: Table S4). ARDS and at-risk patients were otherwise similar in baseline characteristics and biomarker levels.

### Characteristics of glucocorticoid use

Among patients included in the study cohort, 67 (45%) received at least one dose of glucocorticoids between baseline and follow-up samples. Baseline characteristics are provided in Table 1. Patients receiving glucocorticoids were more likely to be female (60% versus 32%, *p =* 0.001), have numerically higher severity of illness scores (median SOFA 8 versus 6.5, *p =* 0.057), and higher plateau pressures (27 [21–29] versus 25 [19–28] cm H2O, *p =* 0.029) compared to those who did not. 25 patients (23%) died at 90 days post-enrollment among patients who did not receive glucocorticoids and 18 (23%) died among patients who received at least one dose (*p =* 0.993, Additional file 1: Figure S1).

**Table 1:** Characteristics of ARDS and at risk from pulmonary insult patients included in the study

Variable	Total cohort (*N* = 148)	No glucocorticoids (*N* = 81)	Glucocorticoids (*N* = 67)	*p*-value
Basic demographics				
Age, years	56.2 (45.4-66.8)	57 (43.9–65.8)	55.9 (47.9–68.1)	0.604
Body mass index	29.4 (25.1–35.8)	30.6 (25.7–35.9)	29 (22.9–34.9)	0.097
Female	66 (45%)	26 (32%)	40 (60%)	0.001
Caucasian	136 (92%)	76 (94%)	60 (90%)	0.282
Comorbidities				
Diabetes mellitus	47 (32%)	27 (33%)	20 (30%)	0.724
Chronic obstructive lung disease	34 (23%)	17 (21%)	17 (25%)	0.56
Congestive cardiac failure	21 (14%)	8 (10%)	13 (19%)	0.154
Chronic renal failure	20 (14%)	7 (9%)	13 (19%)	0.089
Immunosuppression	24 (16%)	5 (6%)	19 (28%)	< 0.001
Chronic liver disease	11 (7%)	8 (10%)	3 (5%)	0.346
Pulmonary fibrosis	5 (3%)	1 (1%)	4 (6%)	0.176
Laboratory findings				
Creatine, mg/dL	1.4 (0.75–2.5)	1.3 (0.7–2.1)	1.6 (0.9–2.6)	0.191
Bicarbonate, mMol/L	24 (21–26.5)	24 (21–27)	24 (20–26)	0.464
Glucose, mg/dL	134 (104–167)	123 (102–149)	140 (118–180)	0.011
White blood cells, × 10^9^/L	11.8 (8–17.2)	13.3 (8.1–16.9)	11.6 (7.4–18.3)	0.662
Hemoglobin, gm/dL	10.4 (9–12.3)	10.7 (9.2–12.7)	10.1 (9–11.8)	0.180
Platelets, × 10^9^/L	176 (123–244)	181 (128–250)	154 (111–235)	0.078
Ventilator parameters				
Tidal volume, mL/kg	6.7 (6–7.9)	6.8 (6–8)	6.6 (6–7.8)	0.392
Positive end expiratory pressure, cm H_2_O	8 (5–10)	8 (5–10)	8 (5–12)	0.299
Plateau pressure, cm H_2_O	25 (19–28)	24 (17–28)	27 (21–29)	0.029
Severity of illness				
SOFA score	7 (5–9)	6.5 (5–8.5)	8 (5–10)	0.057
Acute kidney injury on presentation	73 (49.3%)	36 (44.4%)	37 (55.2%)	0.248
Sepsis on presentation	123 (83%)	64 (79%)	59 (88%)	0.187
Baseline markers of the systemic host immune response				
Angiopoetin-2	9439 (4924–18,646)	8872 (5333–16,341)	10,036 (4321–22,186)	0.629
Interleukin-8	23 (13–43)	22 (13–39)	25 (13–49)	0.225
Interleukin-6	75 (27–232)	83 (33–200)	58 (19–422)	0.474
Procalcitonin	1005 (352–4150)	817 (220–3588)	1232 (507–4510)	0.110
Suppressor of tumorigenicity-2	205,130 (81,454–605,552)	131,909 (68,841–489,845)	270,336 (125,738–740,787)	0.006
Fractalkine	1869 (913–2742)	1210 (797–2157)	2350 (1548–3523)	< 0.001
Interleukin-10	1.3 (0–8.9)	0.8 (0–6.6)	2.9 (0–18.1)	0.031
Pentraxin-3	6418 (2514–14,119)	4571 (2235–9759)	10,147 (2607–22064)	0.017
Soluble receptor for advanced glycation end-products	4422 (2306–8544)	3393 (2279–7288)	5446 (3038–10,697)	0.024
Tumor necrosis factor receptor 1	4576 (2699–8328)	4031 (2632–5796)	5417 (2890–11962)	0.013

Continuous variables are reported as median [interquartile range]

Categorical variables are reported as n (%)

ARDS, acute respiratory distress syndrome; SOFA, Sequential Organ Failure Assessment

SOFA score is modified to exclude the neurologic component

*p* values represent differences between groups by Mann–Whitney U or Fisher’s exact test as appropriate

Notably, patients receiving glucocorticoids had a higher prevalence of pre-existing immunosuppression (28% versus 6%, p < 0.001). In-depth analyses by immunosuppression status were not possible due to sample size limitations, but additional details are provided in Additional file 1: Table S5. Immunosuppressed patients had a lower body mass index and a higher prevalence of chronic renal failure and pulmonary fibrosis compared to those who were not, with higher baseline levels of sRAGE and TNFr1. Average daily dose of glucocorticoids administered to patients with pre-existing immunosuppression in our cohort was similar to the average dose for patients without a history of immunosuppression (*p =* 0.211).

**Fig. 2:**
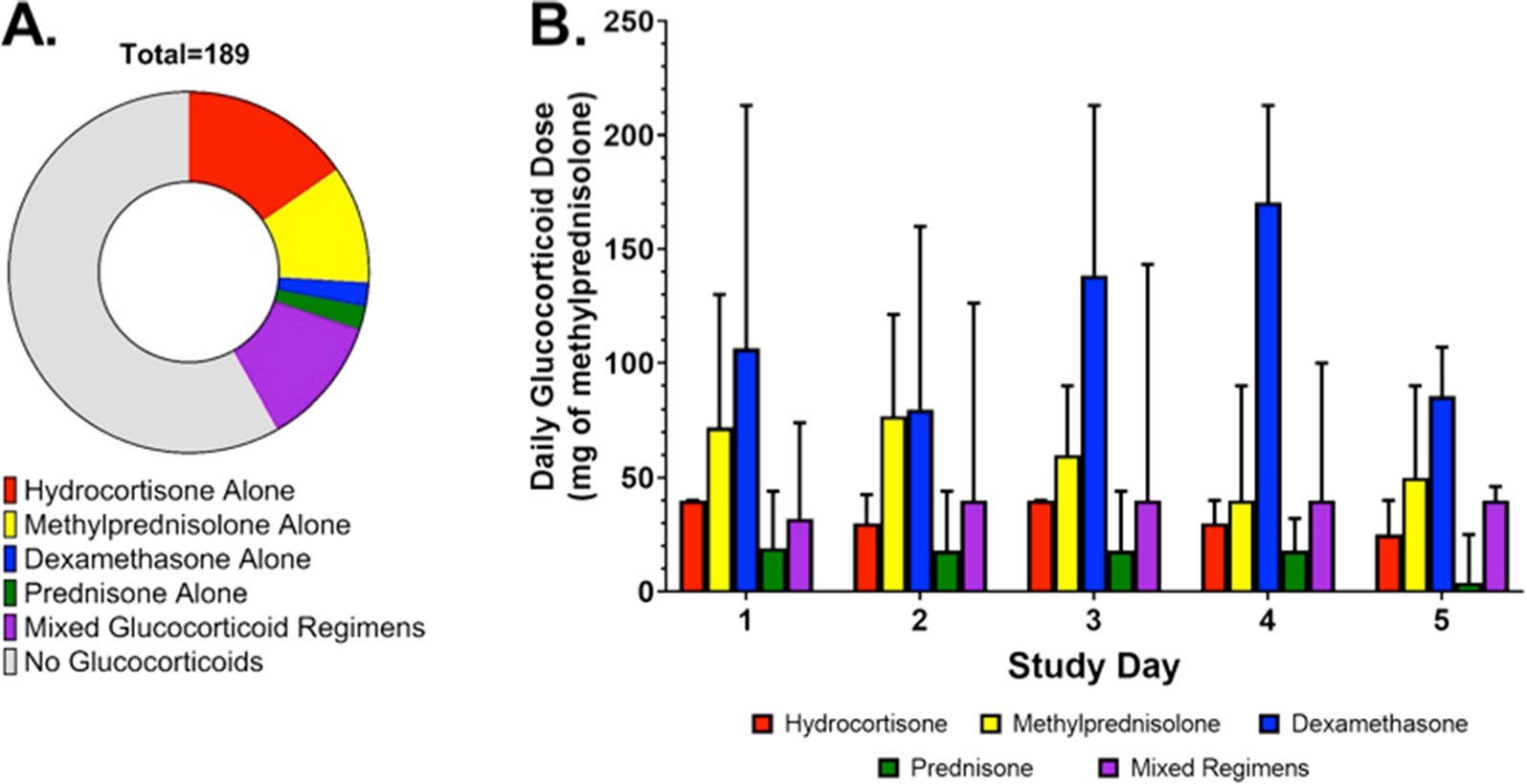
Proportion of study participants receiving glucocorticoids with distribution of type and average daily dose. **A** Illustrates the distribution of glucocorticoids administered to participants between baseline and follow-up samples in the study cohort. Hydrocortisone alone (37%) was the most commonly prescribed glucocorticoid regimen. **B** Illustrates the average dose of glucocorticoids administered per study day grouped by type of glucocorticoid regimen. Data represent median and upper limits of the interquartile range

We next examined the type of glucocorticoids administered to patients between baseline and follow-up samples (Fig. 2A): 25 (37%) received hydrocortisone alone, 18 (27%) received methylprednisolone, 2 (3%) received dexamethasone, and 4 (6%) received prednisone. An additional 18 (27%) received a combination of different types of glucocorticoids between baseline and follow-up samples. Average daily dose of glucocorticoids was equivalent to 40 [21, 67] mg methylprednisolone and appeared to vary based on the type of glucocorticoid administered (Fig. 2B). Most patients received multiple doses of glucocorticoids, only 2 (3%) received a single dose between baseline and follow-up samples. Characteristics of participants receiving different types of glucocorticoids are presented in Additional file 1: Table S6.

### Association of glucocorticoid use with early changes in the systemic host immune response

**Fig. 3:**
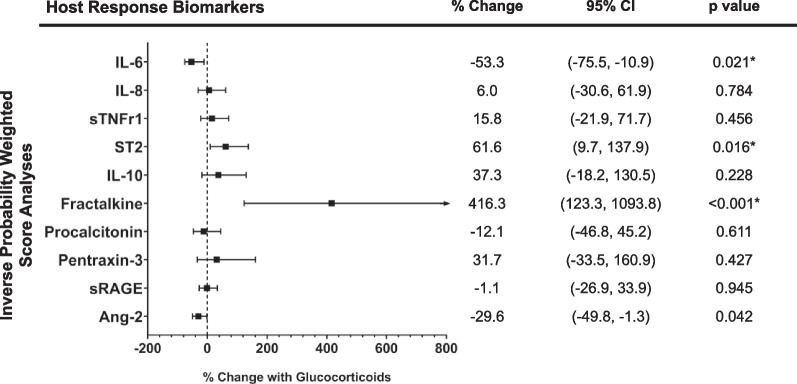
Association of glucocorticoid use with early changes in markers of the systemic host immune response. p-values represent results from doubly robust inverse probability of treatment weighting analysis with each host response biomarker at follow-up as the outcome and with receipt of at least one dose of glucocorticoids between baseline and follow-up samples as the intervention. Unadjusted p-values are reported. *Denotes significance after adjusting for multiple comparisons by the method of Simes with a false discovery rate of 0.1. CI, confidence interval; Ang-2, angiopoietin-2; IL, Interleukin; ST-2, suppressor of tumorigenicity-2; sRAGE, soluble receptor of advanced glycation end-products; sTNFr1, soluble tumor necrosis factor receptor 1

First, we compared markers of the systemic host immune response between glucocorticoid groups at both baseline and at follow-up time points. Baseline levels of ST-2, fractalkine, IL-10, pentraxin-3, sRAGE, and TNFr1 were higher in patients who received at least one dose of glucocorticoids between baseline and follow-up samples compared to patients who did not (Table 1). Next, we determined the association of receipt of at least one dose of glucocorticoids with the systemic host immune response at follow-up in IPTW analyses accounting for propensity to receive glucocorticoids. We found decreases of 53% ([IQR 11–76%], *p =* 0.021) in IL-6 and increases of 416% ([123–1094%], *p* < 0.001) in fractalkine and 61% ([10–138], *p =* 0.016) in ST2 associated with glucocorticoid use compared to no glucocorticoids (Fig. 3). Glucocorticoid use was not significantly associated with changes in other biomarkers after adjusting for multiple comparisons. Follow-up levels of host response biomarkers are presented in Additional file 1: Table S7.

Decreases in IL-6 and increases in fractalkine were similarly observed in sensitivity analyses incorporating decile of propensity score in regression analyses (Model 2, Additional file 1: Table S8). Groups differed in receipt of glucocorticoids prior to baseline samples as 3 (4%) of patients in the No Glucocorticoids and 36 (54%) of patients in the Glucocorticoids group received at least one dose of glucocorticoids prior to the baseline sample (*p* < 0.001). In analyses that additionally adjusted for receipt of glucocorticoids prior to the baseline sample (Models 3 and 4, Additional file 1: Table S8), consistent decreases in IL-6 and increases in fractalkine were observed. However, significant increases in ST-2 that had been observed in Model 1 were not observed in Models 2 through 4. Significant decreases in Ang-2 with glucocorticoid use were observed in Models 2 through 4 but had not been observed in Model 1.

### Host response subphenotypes

Using a previously published parsimonious model [22], 103 patients (70%) were classified into a hypoinflammatory phenotype at baseline, and 45 (30%) patients were classified into a hyperinflammatory phenotype. At baseline, patients in the hyperinflammatory phenotype had higher creatinine on enrollment (2.6 [1.8–3.9] versus 1.1 [0.6–1.7] mg/dL, *p* < 0.001), higher white blood cell counts (15.2 [10.8–20.2] versus 10.5 [7.1–16] × 10^9^/L, *p =* 0.001), and a higher severity of illness (SOFA score 9 [7–11] versus 6 [5–8], *p* < 0.001) compared to hypoinflammatory patients. All measured markers of systemic immune response were higher in hyperinflammatory patients compared to hypoinflammatory patients (Additional file 1: Table S9).

**Fig. 4:**
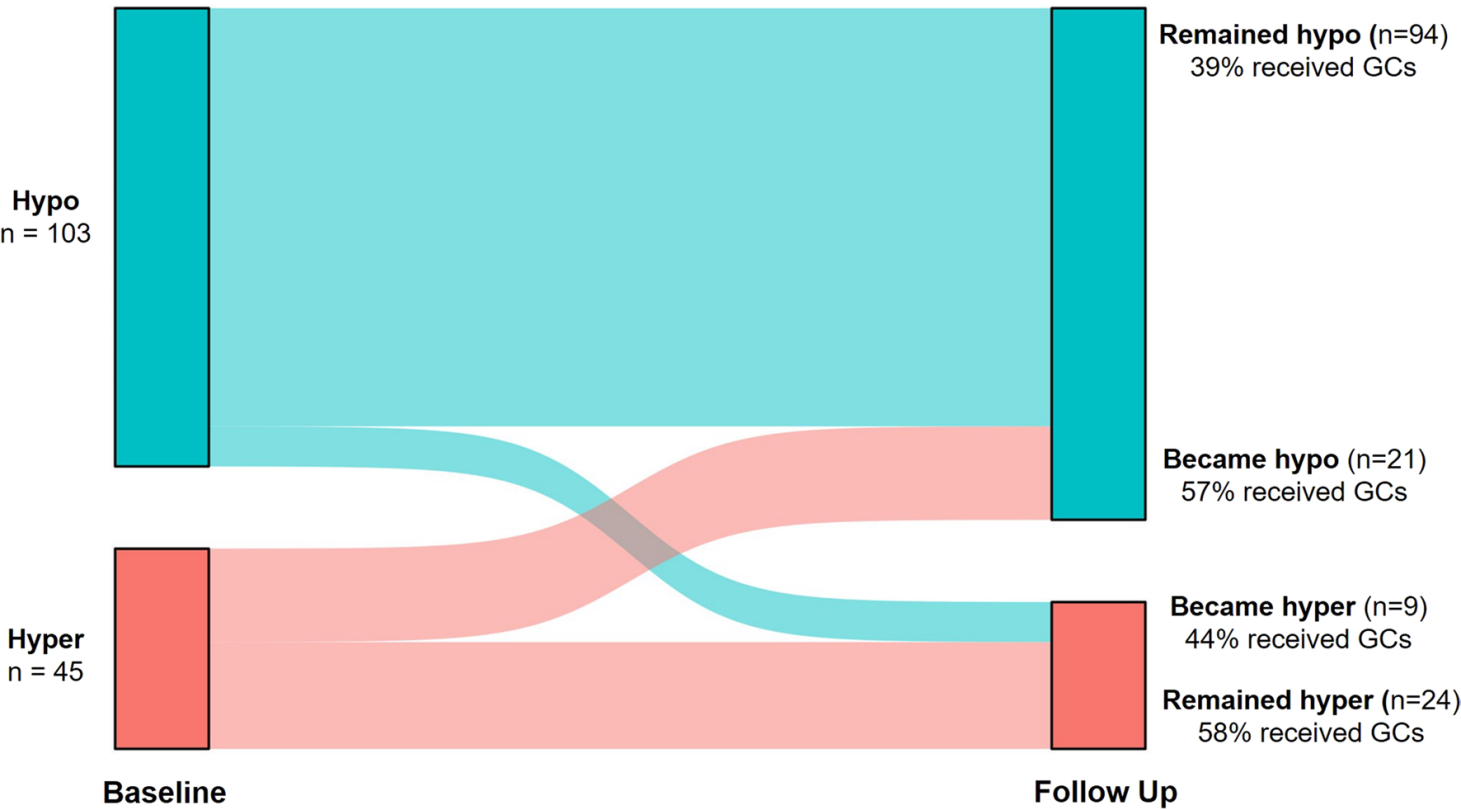
Transition of host response subphenotypes between baseline and follow-up time points. 103 participants (70%) were in the hypoinflammatory subphenotype at baseline. 91% remained hypoinflammatory at follow-up (39% received glucocorticoids) and 9% became hyperinflammatory (44% received glucocorticoids). 45 participants were hyperinflammatory at baseline of whom 53% remained hyperinflammatory (57% received glucocorticoids) and 47% became hypoinflammatory (58% received glucocorticoids). GCs, glucocorticoids; hypo, hypoinflammatory subphenotype; hyper, hyperinflammatory subphenotype

**Fig. 5:**
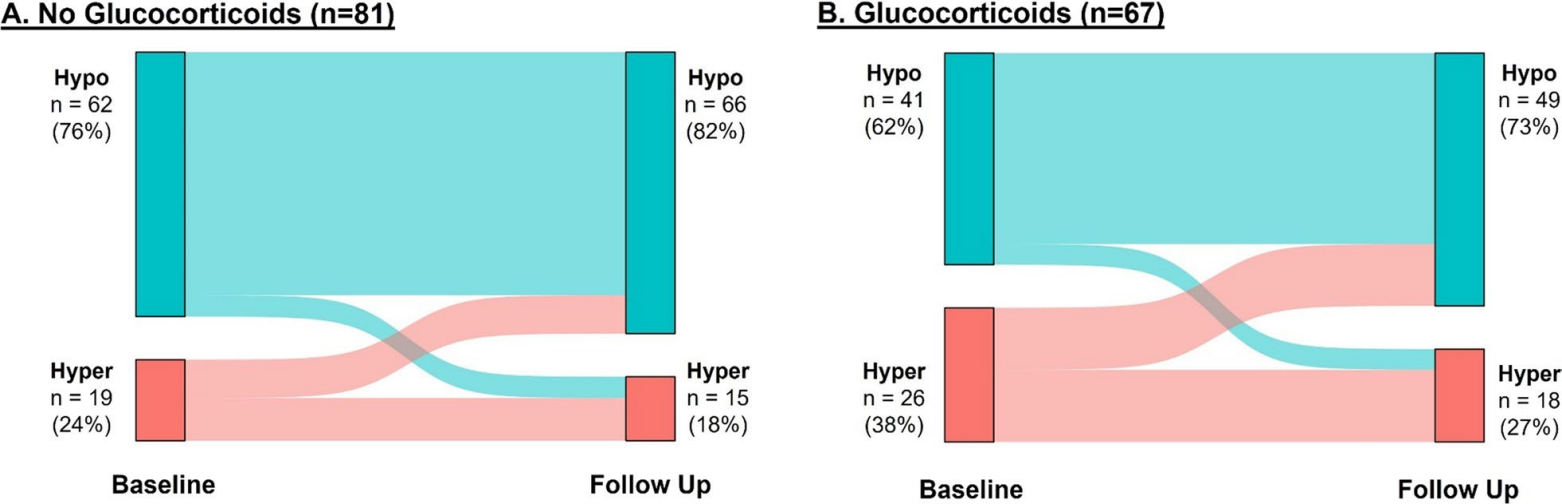
Transition of host response subphenotypes at baseline and follow-up time points stratified by receipt of glucocorticoids. **A** Illustrates the transition of host response subphenotypes in the participants that did not receive glucocorticoids between baseline and follow-up time points. **B** Illustrates the transition of host response subphenotypes in the participants that received at least one dose of glucocorticoids between baseline and follow-up samples. Hypo—hypoinflammatory subphenotype; hyper—hyperinflammatory subphenotype

Glucocorticoid use was higher in hyperinflammatory patients (58% versus 40%, *p =* 0.050). Methylprednisolone (41%) was the glucocorticoid most frequently administered between baseline and follow-up samples to patients in the hypoinflammatory subphenotype, and hydrocortisone (54%) was the glucocorticoid most frequently administered to hyperinflammatory patients. Average daily dose of glucocorticoids was similar between hypoinflammatory (40 [25–67] mg methylprednisolone equivalents) and hyperinflammatory (39 [21–44] mg methylprednisolone equivalents, *p =* 0.116) subphenotypes. Characteristics of patients in host response subphenotypes stratified by receipt of glucocorticoids are presented in Additional file 1: Table S10 and should be interpreted as exploratory due to the sample sizes. The majority of participants remained within the same subphenotype between baseline and follow-up time points (Fig. 4). In exploratory analyses, receipt of glucocorticoids between baseline and follow-up samples was not associated with host response subphenotype at follow-up in the overall cohort (*p =* 0.155), in patients who were hypoinflammatory at baseline (*p =* 0.515), or in patients who were hyperinflammatory (*p =* 0.587) at baseline (Fig. 5). IPTW and propensity score regression analyses by host response subphenotype are presented in Additional file 1: Table S11 and S12 but should similarly be interpreted as exploratory given the smaller sample sizes.

## Discussion

In this study, clinician-guided glucocorticoid use in a cohort of patients requiring mechanical ventilation with ARDS or at-risk due to a pulmonary insult was associated with early changes in the systemic host immune response. At baseline, levels of systemic host immune response biomarkers were higher in patients who received glucocorticoids and thus may have impacted clinical presentation and, indirectly, treatment decisions. We performed IPTW analyses that adjusted for propensity to receive treatment and determined that receipt of glucocorticoids was associated with decreases in circulating IL-6 (an inflammatory cytokine) and increases in fractalkine (an inflammatory chemokine) at an early follow-up time point. Glucocorticoid use may additionally have been associated with decreases in Ang-2 and increases in ST-2, but primary and sensitivity analyses were not consistent, possibly due to sample-size limitations. Prior studies have assessed the effect of methylprednisolone on circulating cytokines in a secondary analysis of a clinical trial in ARDS and, consistent with our studies, demonstrated a decrease in circulating IL-6 [27], which may reflect a decrease in the proinflammatory response. However, we did not observe uniform decreases in proinflammatory biomarkers or increases in anti-inflammatory biomarkers suggesting the effects of real-world use of glucocorticoids on mechanistic pathways may be complex.

Uncertainty remains about the optimal use of glucocorticoids in patients with or at-risk for ARDS. Reconciling discordant results is challenging in the setting of differences in the standardized glucocorticoid regimens used in clinical trials [10]. Our study similarly demonstrates a wide variation in the agents, doses, and duration of glucocorticoids administered to patients with or at-risk for ARDS in our cohort. Our study provides insights into the patients who receive glucocorticoids with higher use in female patients, in immunosuppressed patients, and in patients with higher plateau pressures potentially reflecting a greater severity of lung injury. We acknowledge that the effects of glucocorticoids on mechanistic pathways are ideally interrogated within the context of a randomized clinical trial to reduce the risk of potential confounding present in observational studies. However, as clinician-guided use of glucocorticoids varies from standardized regimens that have previously been used in clinical trials, our analysis provides insights regarding the impact of real-world use of glucocorticoids on host immune response pathways.

Interrogating molecular biomarkers is necessary in informing future prognostic and predictive strategies for management of acute respiratory failure. Protein biomarkers, including angiopoetin-2 and sRAGE, may help identify individuals with acute hypoxic respiratory failure who are at risk for progression to ARDS or to failing non-invasive methods of respiratory support [28–30]. Models that incorporate multiple protein biomarkers with others available may improve risk stratification [18, 20, 22, 31]. Predictive biomarkers that identify potential benefit or harm from a particular treatment are needed to further personalize care and may require baseline or repeated assessments of biomarkers. Recently, a secondary analysis of a randomized clinical trial of immunomodulatory therapy in COVID-19 provided evidence that decreases in IL-6 mediated benefit from treatment and that baseline biomarker levels alone could not similarly predict treatment response [32].

Several studies have suggested response to glucocorticoids may be based on alterations in underlying biologic pathways highlighting a need to understand effects on mechanistic pathways. First, in septic shock, a secondary analysis of the Vasopressin and Septic Shock trial suggested that higher baseline levels of IL-3, IL-6, and CCL4 may identify patients as potential responders to glucocorticoids resulting in reduced mortality [33]. Second, in both pediatric and adult sepsis, transcriptomic signatures of circulating lymphocytes have identified septic endotypes that may experience increased mortality with glucocorticoid use [34–36]. Third, in an observational study of COVID-ARDS, latent class analysis identified hypoinflammatory and hyperinflammatory subphenotypes that potentially differed in response to glucocorticoids with lower mortality in response to glucocorticoids in the hyperinflammatory group and higher mortality in the hypoinflammatory group [37]. Thus, greater understanding of the biologic underpinnings of acute respiratory failure and critical illness may provide insight as to which patients may benefit from glucocorticoids.

In our study, we explored associations of host response subphenotypes with glucocorticoid use. Potentially related to a higher severity of illness, glucocorticoid use was more common in patients in the hyperinflammatory subphenotype at baseline, but small sample sizes prevented IPTW analyses stratified by host response subphenotype or analyses to assess the effects of glucocorticoid use on transitions between subphenotypes. Exploratory analyses did not uncover significant associations between glucocorticoid use and subphenotype classification at an early follow-up time point, but future studies to investigate associations between subphenotypes and glucocorticoid use should be performed in larger cohorts and potentially within the framework of a randomized clinical trial.

Our study has several limitations. First, we specifically selected to include patients that had both baseline and follow-up samples. Thus, our study excludes patients who were transferred out of the ICU or who died prior to follow-up sample collection, thereby potentially introducing selection bias in our cohort. Future studies could address this limitation with more frequent sample collection. Second, the sample size may have limited not only the statistical power to detect smaller differences in circulating biomarkers or clinical outcomes, particularly in analyses focused on subphenotypes but also the ability to investigate differences by dose or type of glucocorticoids. Third, we recognize that patients may have received glucocorticoids for reasons other than directly to treat lung injury (such as for asthma), but we were unable to investigate reasons for glucocorticoid use in our study with enough granularity to make this determination. Fourth, while we used statistical methods to remove some potential confounders, glucocorticoid use was not protocolized, and the intervention modeled in IPTW analyses varied in dose, duration, and type of glucocorticoid administered. Recent studies have highlighted that methods to emulate randomized clinical trial designs from observational data may be less successful when interventions in practice differ from those used in trials [38]. Fifth, while ideally we would have preferred that all baseline samples were collected prior to receipt of any glucocorticoids, we could not due to the observational nature of the study and thus performed sensitivity analyses to ensure consistent results. Sixth, site of infection may influence circulating levels of host response biomarkers in septic patients, and our cohort was heterogenous and included patients with both pulmonary and extrapulmonary sepsis [39]. Proportions of pneumonia were similar between the patients who did and did not receive glucocorticoids; however, unmeasured differences in sites of infection may have impacted biomarker trajectories in our study. Seventh, practice patterns for patients enrolled in the Acute Lung Injury and Biospecimen Repository may potentially differ from the broader population of patients with acute respiratory failure. Big data approaches may better characterize real-world glucocorticoid use but would not have biospecimens available to understand changes in the host response. Lastly, we recognize that our study focused on the impact of glucocorticoids on the systemic host immune response; however, other pathways may be affected such as the adrenocortical axis that could impact clinical outcomes. Indeed, the first studies of heterogeneity of treatment effect of glucocorticoids for sepsis focused on the results of an ACTH stimulation test [40], though results were not replicated in subsequent work [41]. More recent studies have suggested that responders to glucocorticoids in septic shock may be identified through machine learning approaches that incorporate demographics, severity of illness, and circulating cortisol levels [42]. We anticipate that integrative analyses incorporating patient characteristics with analyses of biologic pathways including host response and hormonal pathways will provide greater insight into the responsiveness to glucocorticoid treatment in acute respiratory failure.

## Conclusions

Glucocorticoid use varies in patients with or at-risk for ARDS requiring mechanical ventilation and is associated with early changes in the systemic host immune response. Future studies should continue to explore longitudinal assessments of the systemic host immune response as biomarkers for the response to glucocorticoids in acute respiratory failure.

### Supplementary Information

**Additional file 1:** **Table S1.** Prior studies of glucocorticoid use in patients with acute respiratory distress syndrome (ARDS) or at-risk for ARDS due to severe pneumonia. **Table S2.** Conversion table for glucocorticoids [17]. **Table S3.** Clinical characteristics of ARDS and patients at-risk from a pulmonary insult who were excluded due to biomarker availability compared to patients who were included. **Table S4.** Clinical characteristics comparing patients with ARDS and patients at-risk for ARDS from a pulmonary insult included in the study. **Table S5.** Clinical characteristics comparing patients by pre-existing immunosuppression status. **Table S6.** Clinical characteristics by type(s) of glucocorticoid administered between baseline and follow-up samples. **Table S7.** Systemic host immune response biomarkers measured at follow-up time point. **Table S8.** Sensitivity analyses of the association between glucocorticoid use and systemic host immune response biomarkers at follow-up. **Table S9. **Clinical characteristics by host response subphenotype at baseline. **Table S10. **Clinical characteristics by host response subphenotype and receipt of glucocorticoids. **Table S11.** Sensitivity analyses of the association between glucocorticoid use and systemic host immune response biomarkers at follow-up in the hypoinflammatory phenotype subgroup. **Table S12.** Sensitivity analyses of the association between glucocorticoid use and systemic host immune response biomarkers at follow-up in the hyperinflammatory phenotype subgroup. **Figure S1.** Kaplan–Meier curves for 90-day survival and liberation from mechanical ventilation. Survival curves are adjusted for propensity score. Adjusted hazard ratio for survival (HR 0.96 [95% CI 0.46–2.01], *p =* 0.908) and time to liberation (HR 1.03 [95% CI 0.68–1.54, *p =* 0.905]) did not suggest differences between groups. Hazard ratio generated from Cox proportional hazard modeling with robust regression and proportional hazards assumption tested and not violated in both cases.

## Data Availability

Data and biospecimens for participants in the Acute Lung Injury and Biospecimen Repository are not publicly available. Qualified investigators interested in collaboration may place requests through the Pulmonary Translational Research Core website at: https://paccm.pitt.edu/ptrc/supportrequest.html

## Abbreviations

ARDS: Acute respiratory distress syndrome
COVID-19: Coronavirus disease 2019
ALIR: Acute Lung Injury Registry and Biospecimen Repository
BMI: Body mass index
mSOFA: Modified Sequential Organ Failure Assessment
IL: Interleukin
TNFR1: Tumor necrosis factor receptor 1
ST-2: Suppression of tumorigenicity-2
sRAGE: Soluble receptor for advanced glycation end-products
ang-2: Angiopoietin-2
IQR: Interquartile range
IPTW: Inverse probability of treatment weighting
