# Supplementary material for: Glucocorticoid use in acute respiratory failure from pulmonary causes and association with early changes in the systemic host immune response
Source: Intensive Care Med Exp. 2024 Mar 5;12:24. doi: 10.1186/s40635-024-00605-y (PMC10914652; doi:10.1186/s40635-024-00605-y)
Supplement: Supplementary file 1 — Additional file 1: Table S1. Prior studies of glucocorticoid use in patients with acute respiratory distress syndrome (ARDS) or at-risk for ARDS due to severe pneumonia. Table S2. Conversion table for glucocorticoids [17]. Table S3. Clinical characteristics of ARDS and patients at-risk from a pulmonary insult who were excluded due to biomarker availability compared to patients who were included. Table S4. Clinical characteristics comparing patients with ARDS and patients at-risk for ARDS from a pulmonary insult included in the study. Table S5. Clinical characteristics comparing patients by pre-existing immunosuppression status. Table S6. Clinical characteristics by type(s) of glucocorticoid administered between baseline and follow-up samples. Table S7. Systemic host immune response biomarkers measured at follow-up time point. Table S8. Sensitivity analyses of the association between glucocorticoid use and systemic host immune response biomarkers at follow-up. Table S9. Clinical characteristics by host response subphenotype at baseline. Table S10. Clinical characteristics by host response subphenotype and receipt of glucocorticoids. Table S11. Sensitivity analyses of the association between glucocorticoid use and systemic host immune response biomarkers at follow-up in the hypoinflammatory phenotype subgroup. Table S12. Sensitivity analyses of the association between glucocorticoid use and systemic host immune response biomarkers at follow-up in the hyperinflammatory phenotype subgroup. Figure S1. Kaplan–Meier curves for 90-day survival and liberation from mechanical ventilation. Survival curves are adjusted for propensity score. Adjusted hazard ratio for survival (HR 0.96 [95% CI 0.46–2.01], p = 0.908) and time to liberation (HR 1.03 [95% CI 0.68–1.54, p = 0.905]) did not suggest differences between groups. Hazard ratio generated from Cox proportional hazard modeling with robust regression and proportional hazards assumption tested and not violated in both cases. [file 40635_2024_605_MOESM1_ESM.docx]

**ONLINE DATA SUPPLEMENT**

**Title:** Glucocorticoid Use in Acute Respiratory Failure from Pulmonary Causes and Association with Early Changes in the Systemic Host Immune Response

**Authors:**Nameer Al-Yousif, Seyed M. Nouraie, Matthew J. Broerman, Yingze Zhang, Tomeka Suber, John Evankovich, William Bain, Georgios Kitsios, Bryan J. McVerry, Faraaz A. Shah

**Supplemental Methods**

Review of Risk Factors for Acute Respiratory Distress Syndrome (ARDS): Consensus meetings that determine assignment of participants in the Acute Lung Injury Registry and Biospecimen Repository into clinical groups (ARDS, at-risk for ARDS, not at-risk) include a systematic review of potential risk factors for ARDS as defined by the 2012 Berlin definition [1]. This adjudication includes a review of each participant’s clinical course and available laboratory and microbiologic data to assess presence or absence of direct pulmonary (pneumonia, aspiration, inhalational injury, or lung contusion) and extrapulmonary insults (non-pulmonary sepsis, shock, trauma, transfusion, or pancreatitis) risk factors.

History of Immunosuppression: Patients in the Acute Lung Injury Registry and Biospecimen Repository are classified as having a history of immunosuppression if any of the following criteria are fulfilled: (1) chronic use of glucocorticoids, alkylating agents, antimetabolites, calcineurin inhibitors, mycophenolate, or targeted biologic therapy prior to admission; (2) active chemotherapy against solid or hematologic malignancies prior to admission; or (3) diagnosis of a primary immunodeficiency such as chronic granulomatous disease or common variable immunodeficiency.

**Supplemental Table 1: Prior studies of glucocorticoid use in patients with acute respiratory distress syndrome (ARDS) or at-risk for ARDS due to severe pneumonia**

| Studies in Acute Respiratory Distress Syndrome (ARDS) | | | | |
| --- | --- | --- | --- | --- |
| Study | **Year** | **Setting** | **Glucocorticoid Used** | **Primary Outcome** |
| Bernard et al; PMID: 3317054 [2] | 1987 | Prospective RCT of patients with refractory hypoxemia, diffuse bilateral infiltrates on chest radiography and absence of congestive heart failure documented by pulmonary-artery catheterization. | Methylprednisolone 30 mg/kg predicted body weight every 6 hours for 24 hours | No significant difference in mortality at 45 days or in the reversal of ARDS (p= 0.77). Infectious complications were similar. |
| Meduri et al; PMID: 9669790 [3] | 1998 | Double-blinded RCT of patients with severe ARDS who had failed to improve lung injury score by day 7 in medical ICUs of 4 US medical centers | Methylprednisolone 2 mg/kg predicted body weight daily for 32 days | Reduction in lung injury score, improved PaO2 to FIO2 ratio, higher rates of successful extubation with intervention compared to placebo, with reductions in ICU and hospital-associated mortality. |
| Annane et al; PMID: 16374152 [4] | 2006 | Secondary analysis of a multicenter French RCT of glucocorticoids in septic shock focused on a subset with ARDS | Hydrocortisone 50 mg every 6 hours with fludrocortisone 50 mcg daily | Reduction in mortality in subgroup of patients without a normal response in a cosyntropin stimulation test (hazard ratio 0.57, 95% CI [0.36-0.89]), but no significant difference in patients with a preserved response |
| Steinberg et al, PMID: 16625008 [5] | 2006 | Multicenter ARDSNet trial of persistent ARDS of at least 7 days duration | An initial dose of methylprednisolone 2mg/kg predicted body weight followed by 0.5 mg/kg every 6 hours for 14 days followed by 0.5 mg/kg every 12 hours for 7 days followed by tapering of dose | Similar 60-day mortality compared to placebo control, and higher mortality with methylprednisolone in subgroup with ARDS of at least 14 days duration |
| Meduri et al, PMID: 17426195 [6];  Seam et al, PMID: 21983371 [7] | 2007, 2012 | Multicenter ICU trial of early ARDS in hospitals in Nashville, TN, secondary analysis of biomarkers | Continuous methylprednisolone infusion (1 mg/kg/day) for days 1 to 14 days and then tapered over 2 weeks | Lower lung injury score, lower IL-6, higher protein C, and lower ICU mortality with methylprednisolone compared to placebo |
| Liu et al, PMID: 23158856 [8] | 2012 | ICU study of early ARDS combined with critical illness-related corticosteroid insufficiency in China | Hydrocortisone 100 mg three times daily for seven days | Longer survival time and less incidence of shock with hydrocortisone compared to placebo |
| Tongyoo et al PMID:  27741949 [9] | 2016 | Single-center ICU study in Thailand in adult patients with severe sepsis within 12 hours of meeting ARDS criteria | Hydrocortisone 60 mg every 6 hours for 7 days | Similar 28-day survival compared to placebo control (hazard ratio 0.80, 95% confidence interval [0.46-1.41]), but with improvements in PaO2:FiO2 ratio and in lung injury score in hydrocortisone group |
| Villar et al PMID:  32043986 [10] | 2020 | Multicenter ICU study in Spain in moderate-to-severe ARDS | Dexamethasone 20 mg daily for days 1 to 5, then 10 mg daily for days 6 to 10 | Lower 60-day mortality compared to usual care (36% versus 21%), higher ventilator-free days with dexamethasone |
| Studies in severe pneumonia | | | | |
| Marik et al, 1993; PMID: 8339624 [11] | 1993 | Single-center ICU study in South Africa of patients with severe pneumonia | Single dose of hydrocortisone at 10 mg/kg | No significant difference in TNF-α levels measured 2, 6, or 12 hours after hydrocortisone dose compared to placebo |
| Confalonieri et al; PMID: 15557131 [12] | 2005 | Multicenter ICU study in Italy in patients with severe pneumonia | Hydrocortisone 200 mg bolus followed by infusion of 10 mg/hr for 7 days | Reduction in CRP levels at day 8 as well as higher PaO2:FiO2 ratios and decreased incidence of shock with hydrocortisone compared to placebo |
| Snidjers et al; PMID: 20133929 [13] | 2010 | Single-center study in the Netherlands in hospitalized patients with community-acquired pneumonia; subanalysis in patients with severe pneumonia | Prednisolone 40 mg daily for 7 days | No significant difference between prednisolone and placebo groups in primary outcome of clinical cure by day 7 or in any secondary outcomes. Findings were consistent in the severe pneumonia subgroup. |
| Torres et al; PMID: 20133929 [14] | 2015 | Multicenter trial in Spain in patients with severe community-acquired pneumonia and high CRP | Methylprednisolone 0.5 mg/kg every 12 hours for 5 days | Decrease in primary composite outcome of development of shock, need for new invasive mechanical ventilation, radiographic progression, persistence of respiratory failure, and death (odds ratio 0.34 [95% confidence interval 0.14 to 0.87]) |
| Meduri et al; PMID: 35723686 [15] | 2022 | Multicenter Veterans Affairs trial in patients with severe community-acquired pneumonia | Methylprednisolone 40 mg/day on days 1 through 7, 20 mg per day on days 8 through 14, 12 mg per day on days 15 through 17, and 4 mg per day on days 18 through 20 | No significant difference in 60-day mortality between methylprednisolone and placebo groups, or in any secondary outcomes |
| Dequin et al; PMID: 36942789 [16] | 2023 | Multicenter ICU trial in France in patients with severe pneumonia | Hydrocortisone 50 mg every 6 hours for 4 or 8 days followed by tapering for a total of 8 or 12 days | Lower 28-day mortality with hydrocortisone compared to placebo (6.2% versus 11.9%, p=0.006) |

**Supplemental Table 2: Conversion table for glucocorticoids [17]**

| Glucocorticoid | Equivalent Dose  (mg methylprednisolone) |
| --- | --- |
| Methylprednisolone | 1 |
| Dexamethasone | 5.3 |
| Hydrocortisone | 0.2 |
| Prednisolone | 0.8 |
| Prednisone | 0.8 |

**Supplemental Table 3: Clinical characteristics of ARDS and patients at-risk from a pulmonary insult who were excluded due to biomarker availability compared to patients who were included**

| Variable | Excluded from study (n=187) | Included in study (n=148) | p value |
| --- | --- | --- | --- |
| Basic Demographics | |  |  |
| Age, years | ﻿56 (﻿42-﻿66) | ﻿56 (﻿45-﻿67) | 0.500 |
| Body mass index | 29 (24-36) | 30 (25-36) | 0.651 |
| Gender, Female | 92 (49%) | 66 (45%) | 0.441 |
| Race, Caucasian | 173 (93%) | 136 (92%) | 0.812 |
| Comorbidities |  |  |  |
| Diabetes mellitus | 58 (31%) | 47 (32%) | 0.906 |
| Chronic obstructive lung disease | 36 (19%) | 34 (23%) | 0.420 |
| Congestive cardiac failure | 17 (9%) | 21 (14%) | 0.166 |
| Chronic renal failure | 29 (16%) | 20 (14%) | 0.643 |
| Immunosuppression | 41 (22%) | 24 (16%) | 0.212 |
| Chronic liver disease | 20 (11%) | 11 (7%) | 0.347 |
| Pulmonary fibrosis | 4 (2%) | 5 (3%) | 0.516 |
| Laboratory Findings |  |  |  |
| Creatine, mg/dL | 1.2 (0.8-2.1) | 1.4 (0.8-2.5) | ﻿0.465 |
| Bicarbonate (CO2), mMol/L | 23 (21-27) | 24 (21-27) | ﻿0.834 |
| Glucose, mg/dL | 137 (107-173) | 134 (104-167) | ﻿0.620 |
| White blood cells (WBC), x 10^9^/L | ﻿13 (﻿10-﻿19) | ﻿12 (﻿8-﻿17) | ﻿0.086 |
| Hemoglobin, gm/dL | 10 (9-12) | 10 (9-12) | ﻿0.674 |
| Platelets, x 10^9^/L | 198 (153-265) | 176 (123-244) | ﻿0.015 |
| Ventilator parameters | |  |  |
| Tidal volume, mL | 6.6 (6-7.5) | 6.7 (6-7.9) | ﻿0.635 |
| Positive end expiratory pressure, cmH_2_0 | 8 (5-10) | 8 (5-10) | ﻿0.344 |
| Plateau pressure, cmH_2_0 | 22 (18-25) | 25 (19-28) | ﻿0.002 |
| Severity of illness | |  |  |
| SOFA score | 7 (5-9) | 7 (5-9) | ﻿0.893 |
| Acute kidney injury on presentation | 96 (52%) | 73 (49%) | 0.741 |
| Sepsis on presentation | 141 (75%) | 123 (83%) | 0.106 |
| Baseline markers of the systemic host immune response | | | |
| Ang-2 | 8120 (4328-15474) | 9439 (4924-18646) | ﻿0.190 |
| IL-8 | 19 (9-39) | 23 (13-43) | ﻿0.193 |
| IL-6 | ﻿76.3 (﻿25-﻿278) | ﻿74.6 (﻿27-﻿232) | ﻿0.934 |
| Procalcitonin | 994 (320-3728) | 1005 (352-4150) | ﻿0.638 |
| ST-2 | 197174 (81629-439299) | 205130 (81454-605552) | ﻿0.567 |
| Fractalkine | 1576 (931-2911) | 1869 (913-2742) | ﻿0.652 |
| IL-10 | 1.3 (0-8.16) | 1.3 (0-8.9) | ﻿0.765 |
| Pentraxin-3 | 5152 (2015-13349) | 6418 (2514-14119) | ﻿0.534 |
| sRAGE | ﻿4617 (﻿2211-﻿7247) | ﻿4422 (﻿2306-﻿8544) | ﻿0.722 |
| TNFr1 | 4359 (2377-8445) | 4576 (2699-8328) | ﻿0.687 |
| Host response subphenotype | |  |  |
| Hyperinflammatory subphenotype | 115 (77%) | 103 (70%) | 0.192 |
| Hypoinflammatory subphenotype | 35 (23%) | 45 (30%) |  |
| Glucocorticoid use between baseline and follow-up samples | | | |
| Received at least one dose of glucocorticoids | 81 (45%) | 67 (43%) | 0.415 |
| Average daily dose of glucocorticoids  (mg methylprednisolone) | 36.8 (28.4-45.3) | 40.0 (21.3-62) | 0.995 |

Continuous variables are reported as median [interquartile range]. Categorical bivariables are reported as n (%). p values represent differences between groups by Mann Whitney U or Fisher’s exact test as appropriate. Abbreviations: SOFA- Sequential organ failure assessment; Ang-2- Angiopoietin-2; IL– Interleukin; ST-2 – Suppressor of tumorigenicity-2; sRAGE- Soluble receptor of advanced glycation end-products; TNFr1 - Tumor necrosis factor receptor 1.

**Supplemental Table 4: Clinical characteristics comparing patients with ARDS and patients at-risk for ARDS from a pulmonary insult included in the study**

| Variable | ARDS (n=82) | Pulmonary At-Risk (n=66) | p value |
| --- | --- | --- | --- |
| Basic Demographics | |  |  |
| Age, years | 55.1 (43.4-66.2) | 57 (46.1-69) | 0.234 |
| Body mass index | 29.3 (25.5-34.6) | 29.1 (23.3-35.7) | 0.642 |
| Gender, Female | 45 (54%) | 21 (32%) | 0.012 |
| Race, Caucasian | 76 (92%) | 60 (92%) | 1.000 |
| Comorbidities |  |  |  |
| Diabetes mellitus | 24 (29%) | 23 (35%) | 0.477 |
| Chronic obstructive lung disease | 15 (18%) | 19 (29%) | 0.080 |
| Congestive cardiac failure | 9 (11%) | 12 (18%) | 0.237 |
| Chronic renal failure | 13 (16%) | 7 (11%) | 0.471 |
| Immunosuppression | 20 (24%) | 4 (6%) | 0.003 |
| Chronic liver disease | 8 (10%) | 3 (4%) | 0.348 |
| Pulmonary fibrosis | 5 (6%) | 0 (0%) | 0.053 |
| Laboratory Findings |  |  |  |
| Creatine, mg/dL | 1.4 (0.8-2.4) | 1.4 (0.7-2.6) | 0.817 |
| Bicarbonate (CO2), mMol/L | 24 (21-28) | 24 (21-26) | 0.962 |
| Glucose, mg/dL | 135 (114-156) | 137.5 (103-176) | 0.884 |
| White blood cells (WBC), x 10^9^/L | 11.2 (8.9-17) | 12 (7.4-16.7) | 0.948 |
| Hemoglobin, gm/dL | 10.2 (8.9-12) | 10.9 (9.5-12.7) | 0.053 |
| Platelets, x 10^9^/L | 180 (125.5-261) | 169 (127-226) | 0.300 |
| Ventilator parameters | |  |  |
| Tidal volume, mL | 6.6 (5.8-7.9) | 6.9 (6.1-8.0) | 0.135 |
| Positive end expiratory pressure, cmH_2_0 | 10 (8-14) | 5 (5-10) | <0.001 |
| Plateau pressure, cmH_2_0 | 27 (23-30) | 20 (16-25) | <0.001 |
| Severity of illness | |  |  |
| SOFA score | 7 (5-9) | 7 (5-9) | 0.703 |
| Acute kidney injury on presentation | 38 (46%) | 35 (54%) | 0.408 |
| Sepsis on presentation | 72 (88%) | 51 (78%) | 0.113 |
| Pneumonia | 59 (71%) | 49 (75%) | 0.582 |
| Baseline markers of the systemic host immune response | | | |
| Ang-2 | 8172 (4596-19109) | 9556 (4250-18346) | 0.962 |
| IL-8 | 24 (14-41) | 18 (9-41) | 0.129 |
| IL-6 | 83 (28-227) | 56 (23-181) | 0.327 |
| Procalcitonin | 801 (369-3187) | 1153.6 (254-4348) | 0.654 |
| ST-2 | 174856 (75252-353289) | 333497 (82526-981634) | 0.017 |
| Fractalkine | 1974 (1270-2882) | 1487 (666-2524) | 0.022 |
| IL-10 | 1 (0-10.4) | 1.3 (0-8.7) | 0.477 |
| Pentraxin-3 | 4598 (2528-13692) | 8668 (2280-19202) | 0.448 |
| sRAGE | 5462 (2546-11965) | 3387 (2245-5919) | 0.007 |
| TNFr1 | 4893 (2678-8892) | 3576 (2414-7073) | 0.125 |
| Host response subphenotype | |  |  |
| Hyperinflammatory subphenotype | 28 (34%) | 17 (26%) | 0.208 |
| Hypoinflammatory subphenotype | 55 (66%) | 48 (74%) |  |
| Glucocorticoid use between baseline and follow-up samples | | | |
| Received at least one dose of glucocorticoids | 45 (54%) | 22 (34%) | 0.010 |
| Average daily dose of glucocorticoids  (mg methylprednisolone) | 40 (21-62) | 39 (26-61) | 0.960 |

Continuous variables are reported as median [interquartile range]. Categorical variables are reported as n (%). p values represent differences between groups by Mann Whitney U or Fisher’s exact test as appropriate. Abbreviations: ARDS: acute respiratory distress syndrome; SOFA- Sequential organ failure assessment; Ang-2- Angiopoietin-2; IL– Interleukin; ST-2 – Suppressor of tumorigenicity-2; sRAGE- Soluble receptor of advanced glycation end-products; TNFr1 - Tumor necrosis factor receptor 1.

**Supplemental Table 5: Clinical characteristics comparing patients by pre-existing immunosuppression status**

| Variable | No immunosuppression (n= 124) | Immunosuppression (n=24) | p value |
| --- | --- | --- | --- |
| Basic Demographics | |  |  |
| Age, years | 55.4 (44.5-65.9) | 62.6 (53.7-68.1) | 0.071 |
| Body mass index | 29.9 (25.7-36.4) | 25.1 (22.6-31.2) | 0.009 |
| Gender, Female | 52 (42%) | 14 (58%) | 0.179 |
| Race, Caucasian | 113 (91%) | 23 (96%) | 0.743 |
| Comorbidities |  |  |  |
| Diabetes mellitus | 37 (30%) | 10 (42%) | 0.338 |
| Chronic obstructive lung disease | 29 (23%) | 5 (21%) | 1.000 |
| Congestive cardiac failure | 16 (13%) | 5 (21%) | 0.339 |
| Chronic renal failure | 11 (9%) | 9 (38%) | 0.001 |
| Chronic liver disease | 9 (7%) | 2 (8%) | 1.000 |
| Pulmonary fibrosis | 1 (1%) | 4 (17%) | 0.002 |
| Laboratory Findings |  |  |  |
| Creatine, mg/dL | 1.4 (0.7-2.2) | 1.9 (1.2-3.2) | 0.018 |
| Bicarbonate (CO2), mMol/L | 24 (21-27) | 21.5 (19-25) | 0.044 |
| Glucose, mg/dL | 128 (103-167) | 145.5 (130-170) | 0.148 |
| White blood cells (WBC), x 10^9^/L | 12.1 (8.4-17.1) | 11.3 (5.7-18.8) | 0.403 |
| Hemoglobin, gm/dL | 10.4 (9.2-12.3) | 9.8 (8.5-11.5) | 0.068 |
| Platelets, x 10^9^/L | 180 (127-246) | 148 (105-239.5) | 0.200 |
| Ventilator parameters | |  |  |
| Tidal volume, mL/kg | 6.7 (6-8) | 6.6 (6.2-7.8) | 0.996 |
| Positive end expiratory pressure, cmH_2_0 | 8 (5-10) | 6.5 (5-10) | 0.231 |
| Plateau pressure, cmH_2_0 | 25 (19-28) | 24.5 (19.5-29) | 0.838 |
| Severity of illness | |  |  |
| SOFA score | 7 (5-9) | 7 (6-9) | 0.276 |
| Acute kidney injury on presentation | 58 (47%) | 15 (63%) | 0.185 |
| Sepsis on presentation | 101 (82%) | 22 (92%) | 0.371 |
| Baseline markers of the systemic host immune response | | | |
| Ang-2 | ﻿9429 (﻿4924-﻿18646) | ﻿10798 (﻿4502-﻿18987) | 0.872 |
| IL-8 | ﻿22 (﻿12﻿41) | ﻿27 (﻿15-﻿47) | 0.247 |
| IL-6 | ﻿71 (﻿27-﻿198) | ﻿167 (﻿31-﻿450) | 0.192 |
| Procalcitonin | ﻿938 (﻿286-﻿3828) | ﻿1501 (﻿577-﻿4900) | 0.189 |
| ST-2 | ﻿191599 (﻿73267-﻿622752) | ﻿278269 (﻿157941-﻿391422) | 0.185 |
| Fractalkine | ﻿1723 (﻿826-﻿2678) | ﻿2120 (﻿1849-﻿2876) | 0.064 |
| IL-10 | 1 (0-﻿8.6) | ﻿2.7 (0.8-﻿13.2) | 0.169 |
| Pentraxin-3 | ﻿4729 (﻿2280-﻿12475) | ﻿9608 (﻿4017-﻿19027) | 0.076 |
| sRAGE | ﻿3911 (﻿2259-﻿7590) | ﻿8887 (﻿3665-﻿16490) | 0.001 |
| TNFr1 | ﻿4018 (﻿2579-﻿7367) | ﻿8744 (﻿4684-﻿12428) | <0.001 |
| Host response subphenotype | |  |  |
| Hyperinflammatory subphenotype | 36 (29%) | 9 (37.5%) | 0.469 |
| Hypoinflammatory subphenotype | 88 (71%) | 15 (62.5%) |  |
| Glucocorticoid use between baseline and follow-up samples | | | |
| Received at least one dose of glucocorticoids | 48 (39%) | 19 (79%) | <0.001 |
| Average daily dose of glucocorticoids  (mg methylprednisolone) | 40 (26.1-65.3) | 39 (17.3-48) | 0.211 |

Continuous variables are reported as median [interquartile range]. Categorical variables are reported as n (%). p values represent differences between groups by Mann Whitney U or Fisher’s exact test as appropriate. Abbreviations: ARDS: acute respiratory distress syndrome; SOFA- Sequential organ failure assessment; Ang-2- Angiopoietin-2; IL– Interleukin; ST-2 – Suppressor of tumorigenicity-2; sRAGE- Soluble receptor of advanced glycation end-products; TNFr1 - Tumor necrosis factor receptor 1.

**Supplemental Table 6: Clinical characteristics by type(s) of glucocorticoid administered between baseline and follow up samples**

| Variable | None  (n=81) | Dexamethasone  (n=2) | Hydrocortisone  (n=25) | Methylprednisolone  (n=18) | Prednisone  (n=4) | Mixed  (n=18) |
| --- | --- | --- | --- | --- | --- | --- |
| Baseline demographics | | | | | | |
| Age, years | ﻿57 (﻿43.9-﻿65.8) | 71.2 (69.2-73.2) | 54.6 (47.9-64.5) | 50.7 (33.5-69.4) | 65.6 (62.1-75.9) | 57.9 (53.6-68.1) |
| Body mass index | 30.6 (25.7-35.9) | 30.8 (26.7-34.9) | 29 (23.1-37.3) | 29.7 (25.7-33.3) | 21.3 (20.9-23.4) | 27 (22.9-34.6) |
| Gender, Female | 26 (32.1%) | 2 (100%) | 12 (48%) | 13 (72.2%) | 3 (75%) | 10 (55.6%) |
| Race, Caucasian | 76 (93.8%) | 2 (100%) | 24 (96%) | 15 (83.3%) | 4 (100%) | 15 (83.3%) |
| Comorbidities |  |  |  |  |  |  |
| Diabetes mellitus | 27 (33.3%) | 0 (0%) | 9 (36%) | 5 (27.8%) | 1 (25%) | 5 (27.8%) |
| Chronic obstructive lung disease | 17 (21%) | 1 (50%) | 4 (16%) | 4 (22.2%) | 4 (100%) | 4 (22.2%) |
| Congestive cardiac failure | 8 (9.9%) | 1 (50%) | 6 (25%) | 1 (5.6%) | 3 (75%) | 2 (11.1%) |
| Chronic renal failure | 7 (8.6%) | 1 (50%) | 3 (12%) | 1 (5.6%) | 2 (50%) | 6 (33.3%) |
| Immunosuppression | 5 (6.2%) | 0 (0%) | 5 (20%) | 2 (11.1%) | 2 (50%) | 10 (55.6%) |
| Chronic liver disease | 8 (9.9%) | 0 (0%) | 1 (4%) | 1 (5.6%) | 0 (0%) | 1 (5.6%) |
| Pulmonary fibrosis | 1 (1.2%) | 0 (0%) | 1 (4%) | 0 (0%) | 1 (25%) | 2 (11.1%) |
| Laboratory findings | | | | | | |
| Creatine, mg/dL | 1.3 (0.7-2.1) | 1.35 (0.7-2) | 1.8 (1.2-2.6) | 0.9 (0.6-1.4) | 2.5 (1.3-4.1) | 2.3 (1.5-3.2) |
| Bicarbonate, mMol/L | 24 (21-27) | 22.5 (19-26) | 23 (20-25) | 24.5 (24-29) | 20.5 (16.5-26) | 23.5 (19-28) |
| Glucose, mg/dL | 123 (102-149) | 157 (138-176) | 154 (125-207) | 133.5 (104-145) | 145 (128-171) | 154 (104-185) |
| White blood cells, x 10^9^/L | ﻿13.3 (﻿8.1-﻿16.9) | 8.6 (5.3-11.8) | 15.9 (10.9-21.1) | 9.9 (6.7-13) | 10.4 (3.2-17.9) | 10.5 (5.5-15.3) |
| Hemoglobin, gm/dL | 10.7 (9.2-12.7) | 11.5 (9.9-13) | 9.8 (9.2-11) | 10.1 (9-10.5) | 10.3 (8.8-11.4) | 11.2 (8.2-13.2) |
| Platelets, x 10^9^/L | 181 (128-250) | 104.5 (70-139) | 179 (123-240) | 149.5 (127-219) | 168 (152-221.5) | 139 (104-235) |
| Ventilator parameters | | | | | | |
| Tidal volume, mL/kg | 6.8 (6-8) | 6.7 (6.2-7.2) | 6.6 (6-7) | 6.4 (5.9-7.3) | 8.1 (7.2-8.4) | 6.4 (5.6-8) |
| Positive end expiratory pressure, cmH_2_0 | 8 (5-10) | 6.5 (5-8) | 10 (8-14) | 9 (5-10) | 5 (5-8.5) | 5 (5-12) |
| Plateau pressure, cmH_2_0 | 23.5 (17-27.5) | 23.5 (23-24) | 28 (23-31) | 27 (21-28) | 27 (16-30) | 22 (20-27.5) |
| Severity of illness |  |  |  |  |  |  |
| SOFA score | 6.5 (5-8.5) | 6 (4-8) | 9 (6-11) | 7 (5.5-9) | 6 (5.5-6.5) | 8 (5-10) |
| Acute kidney injury on presentation | 36 (44.4%) | 0 (0%) | 18 (72%) | 5 (27.8%) | 1 (25%) | 13 (72.2%) |
| Sepsis on presentation | 64 (79%) | 2 (100%) | 23 (92%) | 14 (77.8%) | 4 (100%) | 16 (88.9%) |
| Baseline markers of the systemic host immune response | | | | | | |
| Ang-2 | 8872  (5333-16341) | ﻿7827  (﻿5617-﻿10037) | ﻿19097  (﻿7314-﻿30661) | 3634  (1846-9419) | ﻿7725  (﻿4859-﻿10086) | ﻿14645 (﻿8961-﻿21007) |
| IL-8 | 22 (13-39) | ﻿15 (﻿14-17) | ﻿26 (﻿17-﻿59) | 13 (7-28) | 19 (11-28) | 40 (18-228) |
| IL-6 | ﻿83 (﻿33-﻿200) | 46 (28-64) | ﻿142 (﻿32-﻿424) | 19 (5-40) | 39 (3-167) | 399 (36-2042) |
| Procalcitonin | 817 (220-3588) | ﻿2274 (﻿507-﻿4041) | ﻿2235 (﻿984-﻿5110) | ﻿412 (﻿246-﻿772) | ﻿638 (﻿323-﻿1351) | ﻿3698 (﻿1148-﻿4900) |
| ST-2 | 131909  (68841-489845) | ﻿399064 (﻿120286-﻿677841) | ﻿345990 (﻿165987-﻿616103) | 159434  (74966-254605) | ﻿382551 (﻿195818-﻿661685) | 531121  (211553-1355525) |
| Fractalkine | 1210  (797-2157) | ﻿2120  (﻿2120-﻿2120) | ﻿2410  (1635-﻿3053) | ﻿1877  (﻿666-﻿2969) | ﻿1270  (0-﻿1862) | ﻿2879  (﻿2348-﻿4371) |
| IL-10 | 0.8 (0-6.6) | ﻿13.5 (0-27.1) | 4.2 (0-19.1) | 0.7 (0-4.8) | 6.2 (1.5-12) | 7.9 (0.8-28.1) |
| Pentraxin-3 | 4571  (2235-9759) | ﻿11181  (﻿11181-﻿11181) | ﻿11091  (2888-﻿32422) | ﻿7514  (﻿2173-﻿14119) | ﻿4163  (﻿599-﻿13145) | ﻿12939 (﻿4598-﻿48705) |
| sRAGE | ﻿3393  (﻿2279-﻿7288) | ﻿4443  (﻿2260-﻿6626) | ﻿6200  (﻿4834-﻿9743) | ﻿1921  (﻿1320-﻿3851) | ﻿7438  (﻿2693-﻿19882) | ﻿8013  (﻿5446-﻿19018) |
| TNFr1 | 4032  (2632-5797) | ﻿8324  (﻿1863-﻿14786) | ﻿7857  (4688-11962) | ﻿3088  (﻿1760-﻿3920) | ﻿5736  (﻿3370-﻿7450) | ﻿9663  (﻿4705-﻿16273) |

Continuous variables are reported as median [interquartile range]. Categorical variables are reported as n (%). p values represent differences between groups by Mann Whitney U or Fisher’s exact test as appropriate. Abbreviations: ARDS: acute respiratory distress syndrome; SOFA- Sequential organ failure assessment; Ang-2- Angiopoietin-2; IL– Interleukin; ST-2 – Suppressor of tumorigenicity-2; sRAGE- Soluble receptor of advanced glycation end-products; TNFr1 - Tumor necrosis factor receptor 1.

**Supplemental Table 7: Systemic host immune response biomarkers measured at follow up time point.**

| Biomarker | No Glucocorticoids (N=81) | Glucocorticoids (N=67) |
| --- | --- | --- |
| Ang-2 | 7723 (4572-12730) | 7869 (3260-13753) |
| IL-8 | 14 (9-25) | 22 (12-38) |
| IL-6 | 34 (19-92) | 28 (9-94) |
| Procalcitonin | 394 (150-2092) | 584 (193-2928) |
| ST-2 | 55112 (37767-151413) | 145070 (80454-301690) |
| Fractalkine | 1000 (323-1973) | 2134 (1547-3859) |
| IL-10 | 0.6 (0-1.3) | 0.9 (0-9.9) |
| Pentraxin-3 | 3233 (1460-5436) | 6066 (2378-14634) |
| sRAGE | 2659 (1729-3622) | 3622 (2161-7449) |
| TNFr1 | 3946 (2427-6946) | 6626 (3653-11684) |

Host response biomarkers are reported as median [interquartile range]. Abbreviations: Ang-2- Angiopoietin-2; IL– Interleukin; ST-2 – Suppressor of tumorigenicity-2; sRAGE- Soluble receptor of advanced glycation end-products; TNFr1 - Tumor necrosis factor receptor 1.

**Supplemental Table 8: Sensitivity analyses of the association between glucocorticoid use and systemic host immune response biomarkers at follow up**

| Biomarker | % Change for GC | 95% CI Lower Bound | 95% CI Upper Bound | p value |
| --- | --- | --- | --- | --- |
| Ang-2 |  |  |  |  |
| Model 1 | -29.6 | -49.8 | -1.3 | ﻿0.042 |
| Model 2 | -34.0 | -53.4 | -6.6 | ﻿0.019* |
| Model 3 | -41.1 | -61.4 | -10.1 | ﻿0.015* |
| Model 4 | -40.6 | -60.8 | -9.8 | ﻿0.015* |
| IL-8 |  |  |  |  |
| Model 1 | 6.0 | -30.6 | 61.9 | ﻿0.784 |
| Model 2 | -4.7 | -35.6 | 41.1 | ﻿0.808 |
| Model 3 | -5.1 | -36.9 | 42.8 | ﻿0.802 |
| Model 4 | -6.9 | -36.7 | 37.1 | ﻿0.716 |
| IL-6 |  |  |  |  |
| Model 1 | -53.3 | -75.5 | -10.9 | ﻿0.021* |
| Model 2 | -57.8 | -77.1 | -22.2 | ﻿0.006* |
| Model 3 | -66.9 | -83.7 | -32.7 | ﻿0.002* |
| Model 4 | -63.1 | -80.5 | -30.2 | ﻿0.002* |
| Procalcitonin |  |  |  |  |
| Model 1 | -12.1 | -46.8 | 45.2 | ﻿0.611 |
| Model 2 | -28.4 | -55.9 | 16.4 | ﻿0.176 |
| Model 3 | -21.8 | -56.7 | 41.1 | ﻿0.411 |
| Model 4 | -30.9 | -60.7 | 21.4 | ﻿0.197 |
| ST-2 |  |  |  |  |
| Model 1 | 61.6 | 9.7 | 137.9 | ﻿0.016* |
| Model 2 | 45.3 | -0.5 | 112.1 | ﻿0.053 |
| Model 3 | 50.6 | -5.2 | 139.3 | ﻿0.083 |
| Model 4 | 55.7 | -3.4 | 150.9 | ﻿0.069 |
| Fractalkine |  |  |  |  |
| Model 1 | 416.3 | 123.3 | 1093.8 | <0.001* |
| Model 2 | 324.2 | 90.3 | 845.4 | ﻿0.001* |
| Model 3 | 462.5 | 143.2 | 1201.2 | <0.001* |
| Model 4 | 419.8 | 125.1 | 1100.3 | <0.001* |
| IL10 |  |  |  |  |
| Model 1 | 37.3 | -18.2 | 130.5 | ﻿0.228 |
| Model 2 | 23.1 | -23.5 | 98.3 | ﻿0.388 |
| Model 3 | 13.8 | -36.3 | 103.1 | ﻿0.661 |
| Model 4 | 11.5 | -34.8 | 90.6 | ﻿0.689 |
| Pentraxin-3 |  |  |  |  |
| Model 1 | 31.7 | -33.5 | 160.9 | ﻿0.427 |
| Model 2 | 33.6 | -31.5 | 160.5 | ﻿0.393 |
| Model 3 | 35.7 | -42.2 | 218.5 | ﻿0.480 |
| Model 4 | 53.7 | -31.2 | 243.6 | ﻿0.292 |
| RAGE |  |  |  |  |
| Model 1 | -1.1 | -26.9 | 33.9 | ﻿0.945 |
| Model 2 | -5.4 | -28.5 | 25.2 | ﻿0.696 |
| Model 3 | -3.5 | -36.0 | 45.7 | ﻿0.866 |
| Model 4 | 6.8 | -23.6 | 49.4 | ﻿0.697 |
| TNFr1 |  |  |  |  |
| Model 1 | 15.8 | -21.9 | 71.7 | ﻿0.456 |
| Model 2 | 28.1 | -6.7 | 75.8 | ﻿0.122 |
| Model 3 | 17.5 | -24.5 | 83.0 | ﻿0.467 |
| Model 4 | 51.1 | 5.9 | 115.5 | ﻿0.024* |

Model 1 represents the results of the primary analysis of the association between glucocorticoid use and host response biomarkers as assessed by inverse probability of treatment weighting (IPTW) analysis with age, history of immunosuppression, history of chronic obstructive lung disease, severity of illness scores, and vasopressor use at baseline as predictors of glucocorticoid use. Model 2 represents the results of regression analysis with each host response biomarker as the outcome and with adjustment of decile of propensity score. Model 3 repeats the IPTW analysis with adjustment for receipt of glucocorticoids prior to the baseline sample. Model 4 repeats the regression analysis with decile of propensity score with additional adjustment for receipt of glucocorticoids prior to the baseline sample. Unadjusted p-values are reported. * Denotes significance after adjustment for multiple comparisons with the method of Simes with a false discovery rate of 0.1. Abbreviations: SOFA- Sequential organ failure assessment; Ang-2- Angiopoietin-2; IL– Interleukin; ST-2 – Suppressor of tumorigenicity-2; sRAGE- Soluble receptor of advanced glycation end-products; TNFr1 - Tumor necrosis factor receptor 1.

**Supplemental Table 9: Clinical characteristics by host response subphenotype at baseline.**

| Variable | Hypoinflammatory subphenotype  (n=103) | Hyperinflammatory subphenotype  (n=45) | p value |
| --- | --- | --- | --- |
| Basic demographics |  |  |  |
| Age, years | ﻿56.4 (﻿45.3-﻿66.6) | ﻿54.3 (﻿46.3-﻿67.8) | 0.564 |
| Body mass index | 30.2 (25.6-35.8) | 28.3 (23.1-34.6) | 0.250 |
| Gender, Female | 44 (43%) | 22 (49%) | 0.590 |
| Race, Caucasian | 97 (94%) | 39 (87%) | 0.157 |
| Comorbidities |  |  |  |
| Diabetes mellitus | 31 (30%) | 16 (36%) | 0.566 |
| Chronic obstructive lung disease | 20 (19%) | 14 (31%) | 0.139 |
| Congestive cardiac failure | 11 (11%) | 10 (22%) | 0.076 |
| Chronic renal failure | 7 (7%) | 13 (29%) | 0.001 |
| Immunosuppression | 15 (15%) | 9 (20%) | 0.469 |
| Chronic liver disease | 7 (7%) | 4 (9%) | 0.736 |
| Pulmonary fibrosis | 3 (3%) | 2 (4%) | 0.640 |
| Laboratory findings |  |  |  |
| Creatine, mg/dL | 1.1 (0.6-1.7) | 2.6 (1.8-3.9) | <0.001 |
| Bicarbonate, mMol/L | 25 (23-28) | 20 (18-23) | <0.001 |
| Glucose, mg/dL | 133 (104-165) | 140 (103-170) | ﻿0.630 |
| White blood cells, x 10^9^/L | ﻿11 (﻿7-﻿16) | ﻿15 (﻿11-﻿20) | ﻿0.001 |
| Hemoglobin, gm/dL | 11 (9-12) | 10 (8-12) | ﻿0.042 |
| Platelets, x 10^9^/L | 180 (127-246) | 162 (98-242) | ﻿0.270 |
| Ventilator parameters | |  |  |
| Tidal volume, mL/kg | 6.6 (6.0-7.4) | 6.8 (6.2-8.1) | 0.221 |
| Positive end expiratory pressure, cmH_2_0 | 8 (5-10) | 8 (5-12) | ﻿0.885 |
| Plateau pressure, cmH_2_0 | 23 (17-27) | 28 (23-31) | <0.001 |
| Severity of illness | |  |  |
| SOFA score | 6 (5-8) | 9 (7-11) | <0.001 |
| Acute kidney injury on presentation | 43 (42%) | 30 (67%) | 0.007 |
| Sepsis on presentation | 81 (79%) | 42 (93%) | 0.032 |
| Baseline markers of the systemic host immune response | | | |
| Ang-2 | 6744 (3366-11165) | 23012 (15681-38179) | <0.001 |
| IL-8 | 18 (10-30) | 40 (22-77) | <0.001 |
| IL-6 | ﻿51 (﻿19-﻿163) | ﻿193 (﻿43-﻿594) | <0.001 |
| Procalcitonin | 645 (188-1270) | 4900 (2783-5641) | <0.001 |
| ST-2 | 128866 (68841-332271) | 595002 (270336-1335305) | <0.001 |
| Fractalkine | 1306 (797-2221) | 2669 (1988-4371) | <0.001 |
| IL-10 | 0.79 (0-4.8) | 9.3 (0.7-26) | <0.001 |
| Pentraxin-3 | 4431 (2190-11663) | 10686 (4598-32721) | ﻿0.003 |
| sRAGE | ﻿3291 (﻿1997-﻿5649) | ﻿9743 (﻿5185-﻿15905) | <0.001 |
| TNFr1 | 3313 (2255-5074) | 10226 (7206-15302) | <0.001 |
| Glucocorticoid use | |  |  |
| Frequency of glucocorticoid use | 41 (40%) | 26 (58%) | 0.050 |
| Average daily dose, mg methylprednisolone | 40 (25-67) | 39 (21-44) | 0.116 |
| Type of glucocorticoid administered |  |  | 0.012 |
| Dexamethasone | ﻿1 (2%) | ﻿1 (4%) |  |
| Hydrocortisone | ﻿11 (27%) | ﻿14 (54%) |  |
| Methylprednisolone | ﻿17 (41%) | 1 (4%) |  |
| Prednisone | ﻿3 (7%) | 1 (4%) |  |
| Mixed | ﻿9 (22%) | ﻿9 (35%) |  |

Continuous variables are reported as median [interquartile range]. Categorical variables are reported as n (%). p values represent differences between groups by Mann Whitney U or Fisher’s exact test as appropriate. Abbreviations: SOFA- Sequential organ failure assessment; Ang-2- Angiopoietin-2; IL– Interleukin; ST-2 – Suppressor of tumorigenicity-2; sRAGE- Soluble receptor of advanced glycation end-products; TNFr1 - Tumor necrosis factor receptor 1.

**Supplemental Table 10: Clinical characteristics by host response subphenotype and receipt of glucocorticoids.**

| Variable | Hypoinflammatory | | | Hyperinflammatory | | |
| --- | --- | --- | --- | --- | --- | --- |
|  | **No Glucocorticoids  (n=62)** | **Glucocorticoids  (n=41)** | **p- value** | **No Glucocorticoids  (n=19)** | **Glucocorticoids  (n=26)** | **p-value** |
| Basic demographics |  |  |  |  |  |  |
| Age, years | 57.1 (43.9-65.8) | 56 (47.9-68.1) | 0.637 | 56.6 (36.8-68.5) | 54 (49.2-67.8) | 0.629 |
| Body mass index | 31.2 (26.4-36.3) | 29.2 (23.1-34.6) | 0.064 | 28.3 (23.3-32.5) | 28.1 (22.9-36.8) | 0.963 |
| Gender, Female | 17 (27%) | 27 (66%) | <0.001 | 9 (47%) | 13 (50%) | 1.000 |
| Race, Caucasian | 59 (95%) | 38 (93%) | 0.632 | 17 (90%) | 22 (85%) | 1.000 |
| Comorbidities |  |  |  |  |  |  |
| Diabetes mellitus | 19 (31%) | 12 (29%) | 1.000 | 8 (42%) | 8 (31%) | 0.534 |
| Chronic obstructive lung disease | 9 (15%) | 11 (27%) | 0.135 | 8 (42%) | 6 (23%) | 0.206 |
| Congestive cardiac failure | 5 (8%) | 6 (15%) | 0.339 | 3 (16%) | 7 (27%) | 0.481 |
| Chronic renal failure | 3 (5%) | 4 (10%) | 0.432 | 4 (21%) | 9 (35%) | 0.507 |
| Immunosuppression | 4 (7%) | 11 (27%) | 0.008 | 1 (5%) | 8 (31%) | 0.058 |
| Chronic liver disease | 5 (8%) | 2 (5%) | 0.700 | 3 (16%) | 1 (4%) | 0.295 |
| Pulmonary fibrosis | 1 (2%) | 2 (5%) | 0.562 | 0 (0%) | 2 (8%) | 0.501 |
| Laboratory findings |  |  |  |  |  |  |
| Creatine, mg/dL | 1 (0.6-1.6) | 1.1 (0.7-1.7) | 0.573 | 3.4 (1.7-4.5) | 2.5 (1.8-3.3) | 0.269 |
| Bicarbonate, mMol/L | 25 (23-28) | 25 (23-30) | 0.629 | 19 (17-24) | 21 (18-23) | 0.835 |
| Glucose, mg/dL | 122 (103-152) | 138 (120-182) | 0.077 | 128 (88-145) | 154 (114-176) | 0.074 |
| White blood cells, x 10^9^/L | 11 (7.3-16.4) | 10.3 (6.7-14.3) | 0.626 | 15.6 (14-19.1) | 12.1 (10.1-21.1) | 0.377 |
| Hemoglobin, gm/dL | 10.9 (9.3-12.8) | 10.4 (9.6-12.1) | 0.367 | 10.2 (8.3-11.4) | 9.6 (8.5-11.5) | 0.607 |
| Platelets, x 10^9^/L | 187 (139-251) | 151 (123-214) | 0.035 | 160 (120-181) | 167 (91-247) | 0.886 |
| Ventilator parameters |  |  |  |  |  |  |
| Tidal volume, mL/kg | 6.9 (6.0-8.0) | 6.3 (5.9-7.0) | <0.001 | 6.6 (5.7-8.3) | 6.9 (6.4-8.0) | 0.357 |
| Positive end expiratory pressure, cmH_2_0 | 8 (5-10) | 8 (5-12) | 0.594 | 8 (5-10) | 8 (5-14) | 0.254 |
| Plateau pressure, cmH_2_0 | 22 (17-26) | 27 (20-28) | 0.091 | 27 (24-30) | 28 (23-31) | 0.720 |
| Severity of illness |  |  |  |  |  |  |
| SOFA score | 6 (5-8) | 7 (5-9) | 0.048 | 9 (7-11) | 9 (7-11) | 0.778 |
| Acute kidney injury on presentation | 24 (39%) | 19 (46%) | 0.541 | 12 (63%) | 18 (69%) | 0.754 |
| Sepsis on presentation | 47 (76%) | 34 (83%) | 0.466 | 17 (90%) | 25 (96%) | 0.565 |
| Baseline markers of the systemic host immune response | | | |  |  |  |
| Ang-2 | ﻿6974 (﻿4153-﻿11634) | ﻿5460 (﻿2773-﻿10037) | 0.309 | ﻿19484 (﻿15681-﻿36408) | ﻿23039 (﻿15288-﻿43521) | 0.890 |
| IL-8 | ﻿19 (﻿10-31) | 18 (11-29) | 0.904 | ﻿33 (22-54) | 48 (18-228) | 0.198 |
| IL-6 | ﻿71 (﻿31-169) | 35 (13-144) | 0.021 | 190 (41-265) | 341 (43-2042) | 0.270 |
| Procalcitonin | ﻿498 (151-1220) | ﻿730 (﻿264-﻿1496) | 0.370 | 4900 (﻿1588-﻿9097) | ﻿4832 (﻿2783-﻿5284) | 0.469 |
| ST-2 | ﻿107221 (﻿53026-﻿262556) | ﻿191463 (﻿91109-﻿478899) | 0.027 | ﻿526021 (﻿217875-﻿1062704) | ﻿605552 (﻿270336-﻿1470732) | 0.597 |
| Fractalkine | 1042 (704-1904) | ﻿1877 (﻿1270-﻿2524) | 0.006 | ﻿2141 (﻿1568-﻿2669) | ﻿3523 (﻿2187-﻿5005) | 0.012 |
| IL-10 | 0.8 (0-4.1) | 0.8 (0-5) | 0.546 | 4.2 (0-13.2) | 15.9 (0.9-34.6) | 0.043 |
| Pentraxin-3 | ﻿4270 (﻿2190-﻿9615) | ﻿4530 (﻿2241-﻿14119) | 0.379 | ﻿7490 (﻿2405-﻿10817) | ﻿19027 (﻿6175-﻿41260) | 0.065 |
| sRAGE | ﻿3116 (﻿2234-﻿4998) | ﻿3851 (﻿1904-﻿7266) | 0.595 | ﻿8672 (﻿3119-﻿15700) | ﻿12712 (﻿6043-﻿19018) | 0.089 |
| TNFr1 | 3087 (﻿2445-﻿4946) | ﻿3547 (﻿1907-﻿5417) | 0.681 | ﻿8128 (﻿4575-﻿14426) | ﻿12892 (﻿8135-﻿17587) | 0.041 |

Continuous variables are reported as median [interquartile range]. Categorical variables are reported as n (%). p values represent differences between groups by Mann Whitney U or Fisher’s exact test as appropriate. Abbreviations: SOFA- Sequential organ failure assessment; Ang-2- Angiopoietin-2; IL– Interleukin; ST-2 – Suppressor of tumorigenicity-2; sRAGE- Soluble receptor of advanced glycation end-products; TNFr1 - Tumor necrosis factor receptor 1.

**Supplemental Table 11: Sensitivity analyses of the association between glucocorticoid use and systemic host immune response biomarkers at follow up in the hypoinflammatory phenotype subgroup**

| Biomarker | % Change for GC | 95% CI Lower Bound | 95% CI Upper Bound | p value |
| --- | --- | --- | --- | --- |
| Ang-2 |  |  |  |  |
| Model 1 | -33.8 | -49.8 | -12.7 | 0.004 |
| Model 2 | -35.4 | -51.1 | -14.7 | 0.002 |
| Model 3 | -39.1 | -58.0 | -11.7 | 0.009 |
| Model 4 | -38.8 | -57.7 | -11.3 | 0.010 |
| IL-8 |  |  |  |  |
| Model 1 | 4.0 | -24.2 | 42.7 | 0.805 |
| Model 2 | 6.8 | -22.5 | 47.2 | 0.687 |
| Model 3 | 0.0 | -25.6 | 34.4 | 0.999 |
| Model 4 | -0.2 | -26.0 | 34.6 | 0.990 |
| IL-6 |  |  |  |  |
| Model 1 | -37.8 | -61.0 | -0.8 | 0.046 |
| Model 2 | -38.2 | -61.2 | -1.4 | 0.043 |
| Model 3 | -57.5 | -72.5 | -34.3 | <0.001 |
| Model 4 | -57.9 | -73.2 | -33.9 | <0.001 |
| Procalcitonin |  |  |  |  |
| Model 1 | -25.8 | -52.6 | 16.0 | 0.189 |
| Model 2 | -24.2 | -52.1 | 19.7 | 0.233 |
| Model 3 | -24.6 | -55.8 | 28.7 | 0.298 |
| Model 4 | -25.6 | -55.2 | 23.6 | 0.252 |
| ST-2 |  |  |  |  |
| Model 1 | 51.0 | 5.0 | 117.2 | 0.026 |
| Model 2 | 52.5 | 4.2 | 123.3 | 0.030 |
| Model 3 | 46.4 | -2.4 | 119.7 | 0.065 |
| Model 4 | 47.6 | -6.3 | 132.4 | 0.092 |
| Fractalkine |  |  |  |  |
| Model 1 | 521.5 | 161.3 | 1378.2 | <0.001 |
| Model 2 | 588.9 | 186.5 | 1556.8 | <0.001 |
| Model 3 | 650.3 | 231.0 | 1600.7 | <0.001 |
| Model 4 | 704.9 | 215.2 | 1955.3 | <0.001 |
| IL10 |  |  |  |  |
| Model 1 | 84.5 | 16.2 | 192.8 | 0.010 |
| Model 2 | 100.2 | 24.8 | 221.3 | 0.004 |
| Model 3 | 48.4 | -7.6 | 138.2 | 0.101 |
| Model 4 | 73.2 | 3.8 | 189.1 | 0.036 |
| Pentraxin-3 |  |  |  |  |
| Model 1 | 43.0 | -28.5 | 186.2 | 0.309 |
| Model 2 | 55.4 | -24.9 | 221.4 | 0.233 |
| Model 3 | 74.0 | -22.2 | 289.2 | 0.176 |
| Model 4 | 85.1 | -26.0 | 363.0 | 0.186 |
| RAGE |  |  |  |  |
| Model 1 | 2.6 | -19.1 | 30.1 | 0.829 |
| Model 2 | 7.8 | -14.9 | 36.6 | 0.530 |
| Model 3 | 4.2 | -25.7 | 46.2 | 0.808 |
| Model 4 | 12.9 | -17.4 | 54.4 | 0.445 |
| TNFr1 |  |  |  |  |
| Model 1 | 21.7 | -14.0 | 72.3 | 0.173 |
| Model 2 | 35.6 | -1.6 | 86.9 | 0.062 |
| Model 3 | 21.8 | -19.2 | 83.5 | 0.339 |
| Model 4 | 46.0 | 2.1 | 108.6 | 0.038 |

Model 1 represents the results of the primary analysis of the association between glucocorticoid use and host response biomarkers as assessed by inverse probability of treatment weighting (IPTW) analysis with age, history of immunosuppression, history of chronic obstructive lung disease, severity of illness scores, and vasopressor use at baseline as predictors of glucocorticoid use. Model 2 represents the results of regression analysis with each host response biomarker as the outcome and with adjustment of decile of propensity score. Model 3 repeats the IPTW analysis with adjustment for receipt of glucocorticoids prior to the baseline sample. Model 4 repeats the regression analysis with decile of propensity score with additional adjustment for receipt of glucocorticoids prior to the baseline sample. Unadjusted p-values are reported. * Denotes significance after adjustment for multiple comparisons with the method of Simes with a false discovery rate of 0.1. Abbreviations: SOFA- Sequential organ failure assessment; Ang-2- Angiopoietin-2; IL– Interleukin; ST-2 – Suppressor of tumorigenicity-2; sRAGE- Soluble receptor of advanced glycation end-products; TNFr1 - Tumor necrosis factor receptor 1.

**Supplemental Table 12: Sensitivity analyses of the association between glucocorticoid use and systemic host immune response biomarkers at follow up in the hyperinflammatory phenotype subgroup**

| Biomarker | % Change for GC | 95% CI Lower Bound | 95% CI Upper Bound | p value |
| --- | --- | --- | --- | --- |
| Ang-2 |  |  |  |  |
| Model 1 | -13.7 | -45.7 | 37.2 | 0.525 |
| Model 2 | -16.3 | -48.4 | 35.7 | 0.460 |
| Model 3 | -25.6 | -55.1 | 23.1 | 0.242 |
| Model 4 | -12.1 | -51.8 | 60.3 | 0.666 |
| IL-8 |  |  |  |  |
| Model 1 | 17.9 | -50.0 | 178.1 | 0.701 |
| Model 2 | 1.2 | -54.3 | 124.1 | 0.976 |
| Model 3 | -12.0 | -71.5 | 171.3 | 0.820 |
| Model 4 | -10.0 | -72.5 | 194.1 | 0.857 |
| IL-6 |  |  |  |  |
| Model 1 | -69.4 | -87.7 | -23.8 | 0.012 |
| Model 2 | -72.2 | -89.1 | -29.1 | 0.009 |
| Model 3 | -21.0 | -84.4 | 299.5 | 0.771 |
| Model 4 | -36.3 | -86.2 | 195.0 | 0.555 |
| Procalcitonin |  |  |  |  |
| Model 1 | -18.1 | -54.6 | 47.9 | 0.499 |
| Model 2 | -14.4 | -58.5 | 76.6 | 0.666 |
| Model 3 | -23.9 | -59.8 | 44.2 | 0.394 |
| Model 4 | -36.1 | -73.0 | 51.1 | 0.298 |
| ST-2 |  |  |  |  |
| Model 1 | 45.1 | -32.6 | 212.2 | 0.332 |
| Model 2 | 27.6 | -41.7 | 179.5 | 0.533 |
| Model 3 | -10.4 | -67.7 | 148.2 | 0.829 |
| Model 4 | -21.6 | -77.0 | 166.9 | 0.689 |
| Fractalkine |  |  |  |  |
| Model 1 | -1.1 | -69.9 | 225.3 | 0.985 |
| Model 2 | 39.5 | -70.5 | 559.5 | 0.667 |
| Model 3 | 46.1 | -33.4 | 220.8 | 0.336 |
| Model 4 | 121.6 | -65.4 | 1321.0 | 0.391 |
| IL10 |  |  |  |  |
| Model 1 | -25.4 | -73.8 | 113.0 | 0.576 |
| Model 2 | -33.3 | -77.3 | 95.8 | 0.452 |
| Model 3 | 66.4 | -71.1 | 857.1 | 0.560 |
| Model 4 | -10.3 | -87.2 | 530.2 | 0.911 |
| Pentraxin-3 |  |  |  |  |
| Model 1 | -2.6 | -60.3 | 139.4 | 0.954 |
| Model 2 | -14.8 | -71.3 | 152.6 | 0.767 |
| Model 3 | 1.7 | -71.9 | 268.0 | 0.979 |
| Model 4 | 10.4 | -74.3 | 375.2 | 0.891 |
| RAGE |  |  |  |  |
| Model 1 | -19.4 | -46.8 | 22.2 | 0.302 |
| Model 2 | -7.9 | -39.7 | 40.7 | 0.697 |
| Model 3 | 24.0 | -15.2 | 81.5 | 0.260 |
| Model 4 | 18.8 | -27.2 | 93.9 | 0.480 |
| TNFr1 |  |  |  |  |
| Model 1 | 43.0 | -20.9 | 158.7 | 0.190 |
| Model 2 | 126.4 | -0.9 | 417.1 | 0.051 |
| Model 3 | 43.0 | -20.9 | 158.7 | 0.190 |
| Model 4 | 126.4 | -0.9 | 417.1 | 0.051 |

Model 1 represents the results of the primary analysis of the association between glucocorticoid use and host response biomarkers as assessed by inverse probability of treatment weighting (IPTW) analysis with age, history of immunosuppression, history of chronic obstructive lung disease, severity of illness scores, and vasopressor use at baseline as predictors of glucocorticoid use. Model 2 represents the results of regression analysis with each host response biomarker as the outcome and with adjustment of decile of propensity score. Model 3 repeats the IPTW analysis with adjustment for receipt of glucocorticoids prior to the baseline sample. Model 4 repeats the regression analysis with decile of propensity score with additional adjustment for receipt of glucocorticoids prior to the baseline sample. Unadjusted p-values are reported. * Denotes significance after adjustment for multiple comparisons with the method of Simes with a false discovery rate of 0.1. Abbreviations: SOFA- Sequential organ failure assessment; Ang-2- Angiopoietin-2; IL– Interleukin; ST-2 – Suppressor of tumorigenicity-2; sRAGE- Soluble receptor of advanced glycation end-products; TNFr1 - Tumor necrosis factor receptor 1.

**Supplemental Figure 1: Kaplan-Meier curves for 90-day survival and liberation from mechanical ventilation.** Survival curves are adjusted for propensity score. Adjusted hazard ratio for survival (HR 0.96 [95% CI: 0.46-2.01], p = 0.908) and time to liberation (HR 1.03 [95% CI: 0.68-1.54, p=0.905]) did not suggest differences between groups. Hazard ratio generated from Cox proportional hazard modeling with robust regression and proportional hazards assumption tested and not violated in both cases.


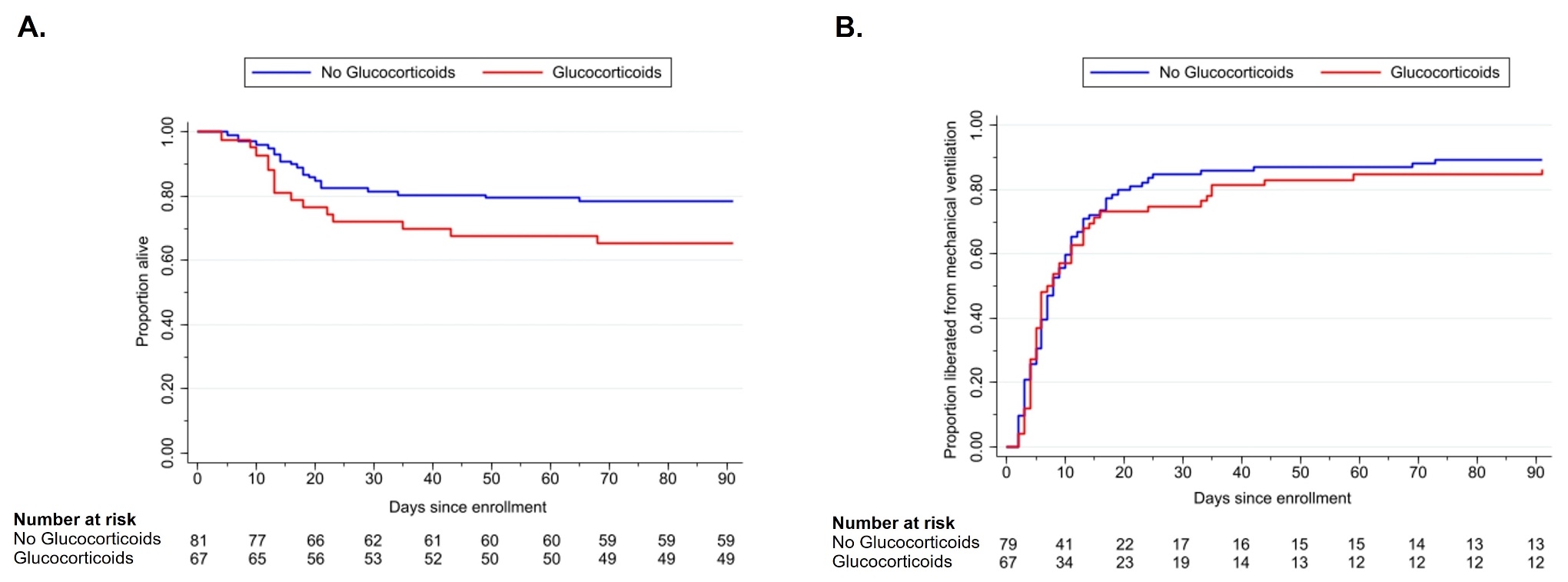


**Supplement References:**

1. Force ADT, Ranieri VM, Rubenfeld GD, Thompson BT, Ferguson ND, Caldwell E, Fan E, Camporota L, Slutsky AS, (2012) Acute respiratory distress syndrome: the Berlin Definition. JAMA 307: 2526-2533

2. Bernard GR, Luce JM, Sprung CL, Rinaldo JE, Tate RM, Sibbald WJ, Kariman K, Higgins S, Bradley R, Metz CA, et al., (1987) High-dose corticosteroids in patients with the adult respiratory distress syndrome. N Engl J Med 317: 1565-1570

3. Meduri GU, Headley AS, Golden E, Carson SJ, Umberger RA, Kelso T, Tolley EA, (1998) Effect of prolonged methylprednisolone therapy in unresolving acute respiratory distress syndrome: a randomized controlled trial. JAMA 280: 159-165

4. Annane D, Sebille V, Bellissant E, Ger-Inf-05 Study G, (2006) Effect of low doses of corticosteroids in septic shock patients with or without early acute respiratory distress syndrome. Crit Care Med 34: 22-30

5. Steinberg KP, Hudson LD, Goodman RB, Hough CL, Lanken PN, Hyzy R, Thompson BT, Ancukiewicz M, National Heart L, Blood Institute Acute Respiratory Distress Syndrome Clinical Trials N, (2006) Efficacy and safety of corticosteroids for persistent acute respiratory distress syndrome. N Engl J Med 354: 1671-1684

6. Meduri GU, Golden E, Freire AX, Taylor E, Zaman M, Carson SJ, Gibson M, Umberger R, (2007) Methylprednisolone infusion in early severe ARDS: results of a randomized controlled trial. Chest 131: 954-963

7. Seam N, Meduri GU, Wang H, Nylen ES, Sun J, Schultz MJ, Tropea M, Suffredini AF, (2012) Effects of methylprednisolone infusion on markers of inflammation, coagulation, and angiogenesis in early acute respiratory distress syndrome. Crit Care Med 40: 495-501

8. Liu L, Li J, Huang YZ, Liu SQ, Yang CS, Guo FM, Qiu HB, Yang Y, (2012) [The effect of stress dose glucocorticoid on patients with acute respiratory distress syndrome combined with critical illness-related corticosteroid insufficiency]. Zhonghua Nei Ke Za Zhi 51: 599-603

9. Tongyoo S, Permpikul C, Mongkolpun W, Vattanavanit V, Udompanturak S, Kocak M, Meduri GU, (2016) Hydrocortisone treatment in early sepsis-associated acute respiratory distress syndrome: results of a randomized controlled trial. Crit Care 20: 329

10. Villar J, Ferrando C, Martinez D, Ambros A, Munoz T, Soler JA, Aguilar G, Alba F, Gonzalez-Higueras E, Conesa LA, Martin-Rodriguez C, Diaz-Dominguez FJ, Serna-Grande P, Rivas R, Ferreres J, Belda J, Capilla L, Tallet A, Anon JM, Fernandez RL, Gonzalez-Martin JM, dexamethasone in An, (2020) Dexamethasone treatment for the acute respiratory distress syndrome: a multicentre, randomised controlled trial. Lancet Respir Med 8: 267-276

11. Marik P, Kraus P, Sribante J, Havlik I, Lipman J, Johnson DW, (1993) Hydrocortisone and tumor necrosis factor in severe community-acquired pneumonia. A randomized controlled study. Chest 104: 389-392

12. Confalonieri M, Urbino R, Potena A, Piattella M, Parigi P, Puccio G, Della Porta R, Giorgio C, Blasi F, Umberger R, Meduri GU, (2005) Hydrocortisone infusion for severe community-acquired pneumonia: a preliminary randomized study. Am J Respir Crit Care Med 171: 242-248

13. Snijders D, Daniels JM, de Graaff CS, van der Werf TS, Boersma WG, (2010) Efficacy of corticosteroids in community-acquired pneumonia: a randomized double-blinded clinical trial. Am J Respir Crit Care Med 181: 975-982

14. Torres A, Sibila O, Ferrer M, Polverino E, Menendez R, Mensa J, Gabarrus A, Sellares J, Restrepo MI, Anzueto A, Niederman MS, Agusti C, (2015) Effect of corticosteroids on treatment failure among hospitalized patients with severe community-acquired pneumonia and high inflammatory response: a randomized clinical trial. JAMA 313: 677-686

15. Meduri GU, Shih MC, Bridges L, Martin TJ, El-Solh A, Seam N, Davis-Karim A, Umberger R, Anzueto A, Sriram P, Lan C, Restrepo MI, Guardiola JJ, Buck T, Johnson DP, Suffredini A, Bell WA, Lin J, Zhao L, Uyeda L, Nielsen L, Huang GD, Group ESS, (2022) Low-dose methylprednisolone treatment in critically ill patients with severe community-acquired pneumonia. Intensive Care Med 48: 1009-1023

16. Dequin PF, Meziani F, Quenot JP, Kamel T, Ricard JD, Badie J, Reignier J, Heming N, Plantefeve G, Souweine B, Voiriot G, Colin G, Frat JP, Mira JP, Barbarot N, Francois B, Louis G, Gibot S, Guitton C, Giacardi C, Hraiech S, Vimeux S, L'Her E, Faure H, Herbrecht JE, Bouisse C, Joret A, Terzi N, Gacouin A, Quentin C, Jourdain M, Leclerc M, Coffre C, Bourgoin H, Lengelle C, Caille-Fenerol C, Giraudeau B, Le Gouge A, Network CR-T, (2023) Hydrocortisone in Severe Community-Acquired Pneumonia. N Engl J Med 388: 1931-1941

17. Czock D, Keller F, Rasche FM, Haussler U, (2005) Pharmacokinetics and pharmacodynamics of systemically administered glucocorticoids. Clin Pharmacokinet 44: 61-98
